# Mitigation of Oxidative Damage by Green Tea Polyphenols and Tai Chi Exercise in Postmenopausal Women with Osteopenia

**DOI:** 10.1371/journal.pone.0048090

**Published:** 2012-10-31

**Authors:** Guoqing Qian, Kathy Xue, Lili Tang, Franklin Wang, Xiao Song, Ming-Chien Chyu, Barbara C. Pence, Chwan-Li Shen, Jia-Sheng Wang

**Affiliations:** 1 Department of Environmental Health Science, University of Georgia, Athens, Georgia, United States of America; 2 Department of Epidemiology and Biostatistics, University of Georgia, Athens, Georgia, United States of America; 3 Department of Mechanical Engineering, Texas Tech University, Lubbock, Texas, United States of America; 4 Laboratory Science and Primary Care, Texas Tech University Health Sciences Center, Lubbock, Texas, United States of America; 5 Department of Pathology, Texas Tech University Health Sciences Center, Lubbock, Texas, United States of America; 6 Laura W. Bush Institute for Women’s Health, Texas Tech University Health Sciences Center, Lubbock, Texas, United States of America; University of Ottawa, Canada

## Abstract

**Background:**

Osteoporosis is a degenerative bone disease predominantly in postmenopausal women. Green tea polyphenols (GTP) and Tai Chi (TC) have been shown to be beneficial on human bone health. This study examined the efficacy of GTP and TC on mitigation of oxidative damage in postmenopausal women with osteopenia.

**Methods:**

A 6-month randomized and placebo-controlled clinical trial was conducted in 171 postmenopausal women with osteopenia, who were recruited from Lubbock County, Texas. These participants were treated with placebo, GTP (500 mg daily), placebo + TC (60-minute group exercise, 3 times/week), or GTP (500 mg daily) + TC (60-minute group exercise, 3 times/week), respectively. Their blood and urine samples were collected at the baseline, 1-, 3- and 6-months during intervention for assessing levels of 8-hydroxy-2′-deoxyguanosine (8-OHdG), an oxidative DNA damage biomarker, and concentrations of serum and urine GTP components.

**Results:**

The elevated concentrations of serum and urinary GTP components demonstrated a good adherence for the trial. A significant reduction of urinary 8-OHdG concentrations was found in all three treated groups during 3-month (*P*<0.001) and 6-month (*P*<0.001) intervention, as compared to the placebo group. The significant time- and dose-effects on mitigation of the oxidative damage biomarker were also found for GTP, TC, and GTP+TC intervened groups.

**Conclusion:**

Our study demonstrated that GTP and TC interventions were effective strategies of reducing the levels of oxidative stress, a putative mechanism for osteoporosis in postmenopausal women, and more importantly, working in an additive manner, which holds the potential as alternative tools to improve bone health in this population.

**Trial Registration:**

ClinicalTrials.gov NCT00625391

## Introduction

Osteoporosis is a degenerative bone disease characterized by low bone mass and structural deterioration of bone tissue. Bone fragility and increased susceptibilities to bone fracture especially in the hip, spine and wrist are common outcomes [1]. Worldwide, osteoporosis is a huge health and social concern as approximately 200 million women suffer from this chronic disease [2]. In the United States, it is estimated that about 44 million people at age of 50 and older suffer from osteoporosis or low bone mass [3]. Postmenopausal women have four times the risk to develop osteoporosis than men due to decreased estrogen levels after menopause and represent the highest risk population [4]. Etiological risk factors associated with osteoporosis include poor nutrition, imbalanced cytokines and hormones, and the aging process [5], [6]. It has been noticed that reactive oxygen species (ROS) plays a key role in the aging process, and contributes greatly to osteoporosis [7]–[9]. Roles of ROS in the pathology of osteoporosis have been reviewed in detail by Manolagas [10], including influences of the generation and survival of osteoclasts, osteoblasts, and osteocytes, disturbance of FoxOs in early mesenchymal progenitors and Wnt signaling pathway, which led to decreased osteoblastogenesis. Excess ROS can damage DNA to form 8-hydroxy-2′-deoxyguanosine (8-OHdG), an oxidative biomarker that has been widely used in human studies to indicate the oxidative stress status [11], [12].

Green tea polyphenols (GTP, extract of green tea) have shown its osteo-protective effects via decreasing oxidative stress, increasing activity of antioxidant enzymes, and decreasing expression of proinflammatory mediators in rodent models [13], and the beneficial effects of GTP on bone health has been reviewed recently [14]. Biomarkers of green tea consumption using green tea components have been validated in human studies [15], and the GTP supplementation has demonstrated chemopreventive effects on cancer [16], cardiovascular diseases [17], and neurodegenerative diseases [18]. However, limited information is available about the beneficial effect of consumption of tea or its bioactive components (i.e., GTP) on bone health in postmenopausal women. On the other hand, Tai Chi (TC) exercise, a form of low to moderate intensity mind-body exercise with aerobic and muscular fitness activity, has been demonstrated to potentially benefit bone health in several human studies [19]–[21]. The potential mechanisms include slowing the decrease of bone mineral density (BMD) [22], reducing the oxidative damage, and enhancing the enzyme activity of superoxide dismutase (SOD) [23]. Nevertheless, the information on effects of GTP and TC, especially their combined effects, on oxidative stress status of postmenopausal women is lacking.

In this study, a 6-month placebo-controlled randomized trial was conducted to examine the pharmacokinetics of GTP and effects of GTP and TC on mitigation of the oxidative damage biomarker in 171 study participants, who were recruited from a large community pool including 1,065 postmenopausal women. As for the whole project of this clinical trial, the detailed study protocol [24], the data on the safety issues and life quality of study participants [25], and clinical outcomes on bone health [26] have been published previously.

## Methods

The protocol for this trial and supporting CONSORT checklist are available as supporting information; see Checklist S1 and Protocol S1.

### 1. Study Participants and Design

**Figure 1:**
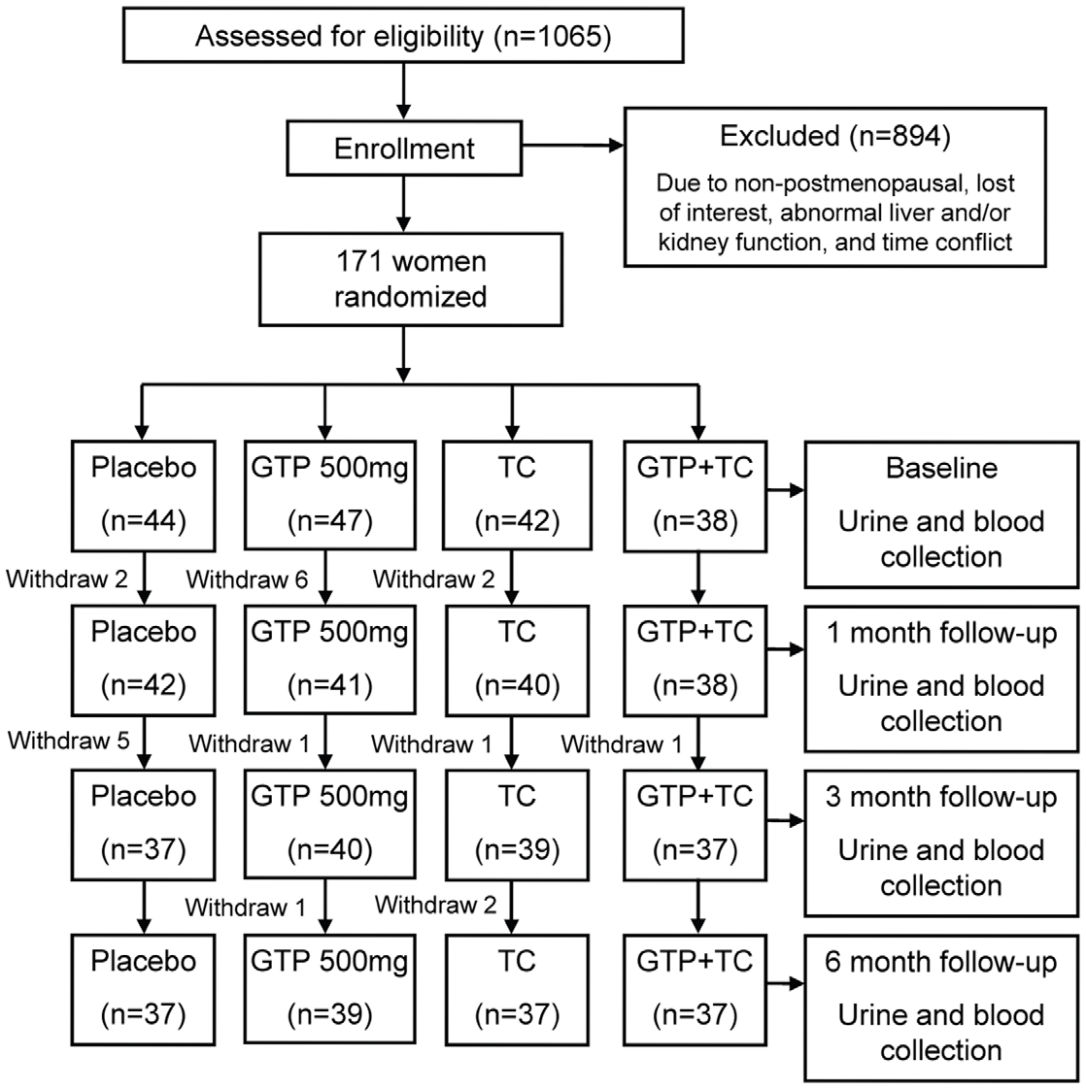
Flow chart of the trial.

The detailed flow of the trial is described in Figure 1. The study participants were screened and enrolled in 2007 from Lubbock, Texas and the surrounding area. The trial was conducted in 2007–2008. The study followed the randomized and placebo-controlled clinical trial guidelines from National Institute of Health (NIH). Briefly, a total of 171 community-dwelling postmenopausal women were recruited to participate in this 6-month trial. The recruited participants were randomized in a stratified method based on age (≥65 or <65 years old), history of green tea consumption, and history of mind-body exercise and assigned to one of the four treatment groups: Placebo group (age: 57.6 (7.5), mean (SD)): medicinal starch 500 mg daily; GTP group (age: 56.5 (5.5), mean (SD)): GTP 500 mg daily; Placebo+TC group (age: 58.3 (7.7), mean (SD)): medicinal starch 500 mg daily and 24-move simplified Yang-style TC training (60 minutes per session, 3 sessions per week), and GTP+TC group (age: 57.6 (6.7), mean (SD)): GTP 500 mg daily and 24-move simplified Yang-style TC training (60 minutes per session, 3 sessions per week). A daily dose of GTP or placebo material was administered as two capsules (250 mg each). Daily consumption of such a GTP dose (500 mg) was equivalent to 4 cups of green tea (about 500 mL). During intervention, all participants were provided with 500 mg elemental calcium and 200 IU vitamin D (as cholecalciferol) daily. The complete study protocol has been reported in detail previously [24]. Inclusion criteria in this study included: postmenopausal women (at least 2 years after menopause) with osteopenia (mean lumbar spine and/or hip BMD T-score between 1 and 2.5 standard deviation (SD) below the normal sex-matched areal BMD of the reference database; normal function of thyroid, liver, and kidney; and serum 25-hydroxy vitamin D (25-OH-D, ≥20 ng/mL)). Women were excluded if they had a disease condition or were taking medication known to affect bone metabolism; history of cancer except for treated superficial basal or squamous cell carcinoma of the skin; uncontrolled intercurrent illness or physical condition that would be a contraindication to exercise; depression; cognitive impairment; and if unwilling to accept randomization. Written informed consent was obtained from all the participants before enrollment and the study was approved by the Texas Tech University Health Sciences Center Institutional Review Board.

### 2. Randomization and Blinding

A central allocation schedule for randomization was prepared through a computer-generated random sequence of the four treatment allocations. Treatment assignments were then made from separate randomization sequences created for each stratum. Placebo or GTP capsules dispersion was conducted by the site research pharmacist according to the patient’s randomization assignment. Research staff and medical staff were unaware of the treatment assignment. Sample analysis, data collection and statistical analysis were blinded to the technical personnel.

### 3. Sample Size

Data from previous studies were used for calculation of sample size. The effect size of 0.75 was assumed based on the baseline mean and standard deviation (SD) values for the primary outcome of urinary 8-OHdG (7.5±2.5 pmol/mL) [27]. Equal sample size for each group was assumed. The main comparisons are expected to be made as follows: placebo vs. GTP; placebo vs. placebo+TC; and placebo vs. GTP+TC. The sample size was calculated based on detecting a 25% change in urinary 8-OHdG, as compared to placebo group, with a power of 0.8 at α = 0.05. An assumed correlation coefficient of 0.80 between baseline and follow-up measurements within a treatment group was used. The sample size for each group was estimated to be 32 for urinary 8-OHdG based on a previously reported method [28]. Therefore, a total sample size of 128 participants is needed which requires 151 participants at an attrition rate of 15%.

### 4. Chemicals and Instruments

GTP capsules and placebo agents were supplied by Zhejiang Yixin Pharmaceutical Co., Ltd. China (GTP IND no. 77,470 by FDA of USA). The main GTP components were 46.5% of epigallocatechin-3-gallate (EGCG), 21.25% of epigallocatechin (ECG), 10% of epicatechin (EC), 7.5% of epicatechin-3-gallate (EGC), 9.5% of gallocatechingallate (GCG), and 4.5% of catechin with the purity higher than 98.5%. Authentic standards of EC, ECG, EGC, and EGCG, beta-glucuronidase, sulfatase, and tetrahydroxyfuran (THF) were purchased from Sigma Chemical Co. (St. Louis, MO). Acetonitrile (ACN) was purchased from Burdick & Jackson (Muskegon, MI). Ethyl acetate, methylene chloride, and sodium dihydrophosphate (NaH_2_PO4) were products of Fisher Scientific (Pittsburgh, PA). An ESA HPLC-CoulArray system (Chelmsford, MA) was used for detection of GTP components and metabolites as well as urinary 8-OHdG.

### 5. Blood and Urine Sample Collection and Processing

All participants reported to Clinical Research Center at Texas Tech University Medical Center for blood/urine collection. The blood samples were collected between 7∶00 and 10∶00 a.m. after an overnight fast with abstinence of food, drink, nicotine, and caffeine. Blood was drawn from a superficial arm vein with a syringe, transferred to a vacutainer, allowed to clot at room temperature, and then centrifuged at 1500 g for 10 min within 2 hours of collection to separate plasma or serum. Urine samples were also collected in acid-washed polyethylene containers. After the total volume of the urine sample was measured to an accuracy of 0.1 ml and recorded, urine was aliquoted. All plasma and urine samples were stored at −80°C before analyses.

### 6. Extraction of Serum and Urinary GTP

The protocol for serum GTP extraction was previously published [15]. Briefly, 200 µL serum samples were incubated with beta-glucuronidase (500 units) and sulfatase (20 units) at 37°C for 45 min to release the conjugated GTP components before repeated extraction with 400 µL methylene chloride to remove proteins and lipids. The aqueous phases were pooled for twice extraction with 700 µL ethyl acetate, and the organic phase was vacuum-dried with a Labconco Centrivap concentrator (Kansas City, MO) and reconstituted for HPLC-ECD analysis.

Urinary GTP extraction followed a previously established protocol [27]. Briefly, thawed urine samples were centrifuged and 1 mL supernatant was taken for 60 min incubation with beta-glucuronidase (500 units) and sulfatase (2 units) at 37°C to release conjugated GTP, then directly extracted twice with ethyl acetate. Organic phases were pooled, dried *in vacuo*, and reconstituted in 15% acetonitrile before analysis.

### 7. HPLC-ECD Analysis of Serum and Urinary GTP Components

The method for analyzing serum and urinary GTP conjugates was modified from previously established protocols [27]. GTP analysis was conducted in the ESA HPLC-CoulArray system (Chelmsford, MA). The mobile phases consisted of buffer A (30 mM NaH_2_PO_4_:ACN:THF at 98%: 1.8%: 0.2%, v/v, pH 3.35) and buffer B (15 mM NaH_2_PO_4_:ACN:THF at 30%: 63%: 7%, v/v, pH 3.45). The Zorbax Eclipse XDB-C18 (5 micron, 4.6×250 mm) was used and maintained at 35°C during separation. Flow rate was set at 1 mL/min and a gradient was generated to separate the GTP components within 60 min. The 8 channels of the CoulArray detector were sequentially set at −90, −10, 70, 150, 230, 310, 380 and 450 mV potentials for the detection of GTP components. The main peaks appeared at −10 mV (EGC), 70 mV (EC, EGCG), and 150 mV (ECG). Calibration curves for individual standard GTP component were generated separately, and EGC, EC, EGCG, and ECG were eluted at around 14, 21, 24, and 29 min, respectively. Quality assurance and quality control procedures included analysis of one authentic standard for every set of five samples and simultaneous analysis of a quality control sample daily. The limits of detection were 1.0 ng/mL urine for EC and EGC and 0.5 ng/mL serum for EGCG and ECG, respectively. Urinary GTP components were adjusted by creatinine level to eliminate the variation in urine volume. Urinary creatinine concentration was determined colorimetrically with a Diagnostics Creatinine Kit (Sigma Co).

### 8. Measurement of Urinary 8-OHdG

The procedure for 8-OHdG analysis was modified from established protocols [27]. Urinary 8-OHdG was extracted from 1 mL urine with the Oasis® HLB 3 cc (60 mg) cartridge. The eluents were dried under ultra-pure N_2_ stream and reconstituted in buffer (10 mM ammonium acetate in 2% MeOH, pH 4.3) for analysis with the ESA HPLC-CoulArray system. The HPLC column for 8-OHdG analysis was Waters YMC basicTM column (S3 µm, 4.6×150 mm). The mobile phases consisted of buffer A (10 mM ammonium acetate, pH 4.3) and buffer B (100% methanol). Flow rate was kept at 0.8 mL/min and a linear gradient (0–40% MeOH in 15 min) was applied for chromatographic separation with the peak of 8-OHdG eluted at around 9.5 min. The CoulArray Detector was set at 270, 300, 330, and 360 mv, with the highest peak appeared at 330 mv channel. Authentic standard 8-OHdG was used for qualification by retention times and response patterns, and quantification by calibration curves. Quality assurance and quality control procedures included analysis of one authentic standard for every set of five samples and simultaneous analysis of a spiked urine sample daily. The limit of detection for 8-OHdG was 1 ng/mL urine. The amount of 8-OHdG was adjusted by urinary creatinine concentrations, which was also determined with a Diagnostics Creatinine Kit (Sigma Co).

### 9. Statistical Analysis

All of the data generated were stored in an Excel database and analyzed with SAS software version 9.3 (SAS Institute Inc., Cary, NC). Median, mean, standard deviations (SD) and range were calculated for concentrations of 8-OHdG, EC and EGC in urine, and EGCG and ECG in serum. The values were expressed as median and mean ± SD unless otherwise stated. To assess the efficacy of GTP, TC, or GTP+TC intervention arms, the statistical evaluation focused on the comparisons among different treatment arms and different time points. For the parameters that were normally distributed (serum EGCG and ECG), repeated measures ANOVA and Bonferroni correction were used to compare differences among means of different times separately for serum EGCG and ECG. For the parameters that were not normally distributed (urinary EC, EGC and 8-OHdG), the Kruskal-Wallis test of one-way ANOVA followed by Wilcoxon rank sum test with Bonferroni correction was used to compare the differences among different treatment groups; and the Friedman’s test followed by Wilcoxon signed rank test with Bonferroni correction was used to compare the differences among different time points (urinary EC, EGC and 8-OHdG) given the dependence of measurements. For urinary EC and EGC, the test of difference between GTP and GTP+TC groups at the same time point was done by Wilcoxon rank sum test with Bonferroni correction. To evaluate the effect of dose and time interactions on treatment arms, a nonparametric model for analysis of repeated measurements was applied as previously described [29]. A *P*-value of less than 0.05 (two-tailed) was considered statistically significant.

## Results

### 1. Overall Study Outcome

A total of 88% (150/171) participants completed for this 6-month clinical trial with complete data. Seven (16%) participants in the Placebo arm, 5 (12%) in the Placebo+TC arm, 8 (17%) in the GTP arm, and 1 (3%) in the GTP+TC arm withdrew before the end of the study due to accidental fall at home (1 participant), relocation (2 participants), time conflicts (6 participants), lost to follow-up (5 participants) and lost interest (7 participants). The compliance rate was 89% for both GTP and placebo capsules and the adherence rate for TC classes was 83%.

### 2. Serum GTP in Study Participants

**Figure 2:**
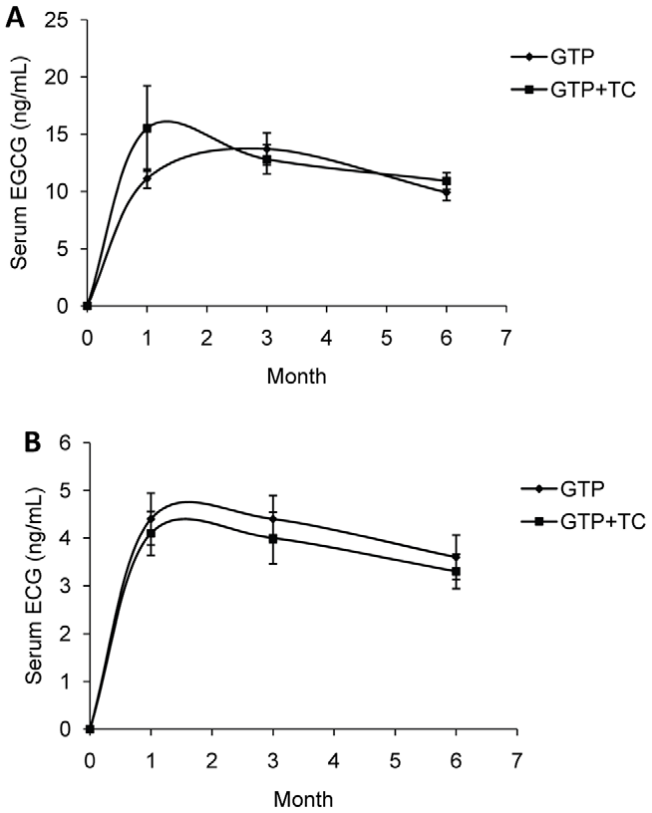
Mean (±SEM) serum GTP concentrations in study participants in the clinical trial. A: serum EGCG concentrations; B: serum ECG concentrations. Starting from first month, serum EGCG and ECG kept constant with minor fluctuations in the GTP and GTP+TC groups. The differences among different time points (1-, 3-, or 6-months) within each group and between groups were tested with repeated measures ANOVA followed by Bonferroni correction for multiple comparisons for each outcome and the significant difference was found in EGCG concentrations in the GTP group between 3- and 6-months (*P*<0.050). No significant differences were found between these two groups at each time point (*P*>0.050).

To examine the adherence and compliance of study participants in this intervention trial, serum and urinary GTP concentrations were measured at different intervention intervals, 0, 1-, 3- and 6- month. There were no detectable GTP components in the placebo group and TC group over 4 collection times of serum samples. There were no GTP components in the baseline of all 4 treatment groups, which was consistent with the questionnaire data, i.e., no green tea drinking habits in these study participants. Among GTP-intervention groups (GTP and GTP+TC), only EGCG and ECG were constantly detectable in serum samples of the study participants after intervention. Serum EGCG concentrations in both GTP and GTP+TC groups were elevated in study participants at 1-month after intervention and kept at the similar levels at 3- and 6-months (Figure 2A). No significant time effect on serum EGCG concentrations was found in GTP+TC Group *P* = 0.413) while significant time effect were found in GTP group (*P* = 0.019) and between 3- and 6-months (*P*<0.050). Serum ECG concentrations in both GTP and GTP+TC groups were also elevated in study participants at 1-month after the intervention and kept at similar concentrations at 3- and 6-months (Figure 2B.). No statistically significant time effect on serum ECG concentrations was found (*P* = 0.321 in GTP group and *P* = 0.438 in GTP+TC group), though there were some fluctuations in data between 3-month and 6-month time points.

### 3. Urinary GTP in Study Participants

**Figure 3:**
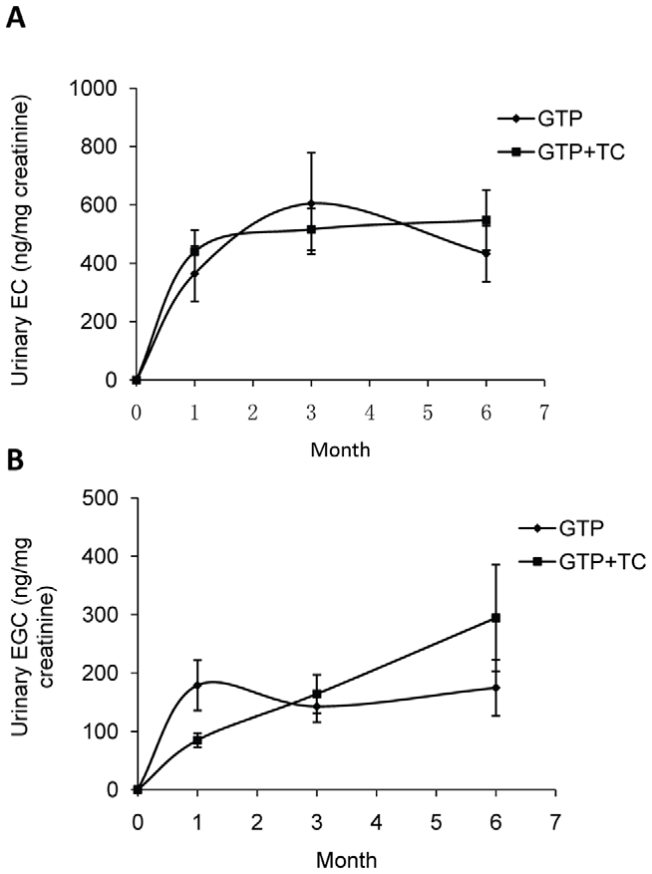
Mean (±SEM) urinary GTP concentrations in study participants in intervention groups. A: urinary EC concentrations; B: urinary EGC concentrations. Starting from first month, urinary EC and EGC concentrations kept constant with minor fluctuations in GTP and GTP+TC groups. No significant differences were found between these two groups at same time points (*P*>0.050, tested with Wilcoxon rank sum test). The differences among differnt time points within each group were tested with Friedman’s test followed by Wilcoxon signed rank test with Bonferroni correction for multiple comparisons for each outcome and the significant difference only existed in the EGC concentrations in GTP+TC group between 1- and 3-months (*P*<0.050).

There were no detectable green tea polyphenol components in the placebo group and TC group participants over 4 time-points collected urine samples. Among GTP intervention groups (GTP and GTP+TC), only EC and EGC were constantly detectable in urinary samples of the study participants. As shown in Figure 3A, urinary EC concentrations in both GTP and GTP+TC groups were elevated in study participants at 1-month after the intervention and kept at higher concentrations at 3- and 6-months. No significant time effect on urinary EC concentrations was found (*P* = 0.201 in GTP group and *P* = 0.575 in GTP+TC group). Urinary EGC concentrations in GTP-intervened group were elevated in study participants at 1-month after the intervention and kept at constantly higher concentrations at 3- and 6-months (Figure 3B), whereas urinary EGC concentrations in GTP+TC group were kept elevated linearly in study participants in 1-, 3-, and 6-months. No significant time effect on urinary EGC concentrations was found in GTP group (*P* = 0.689), while significant difference occurred in GTP+TC group (*P* = 0.035) with difference found between 1- and 3-months.

### 4. Urinary 8-OHdG Concentrations in Study Participants

**Figure 4:**
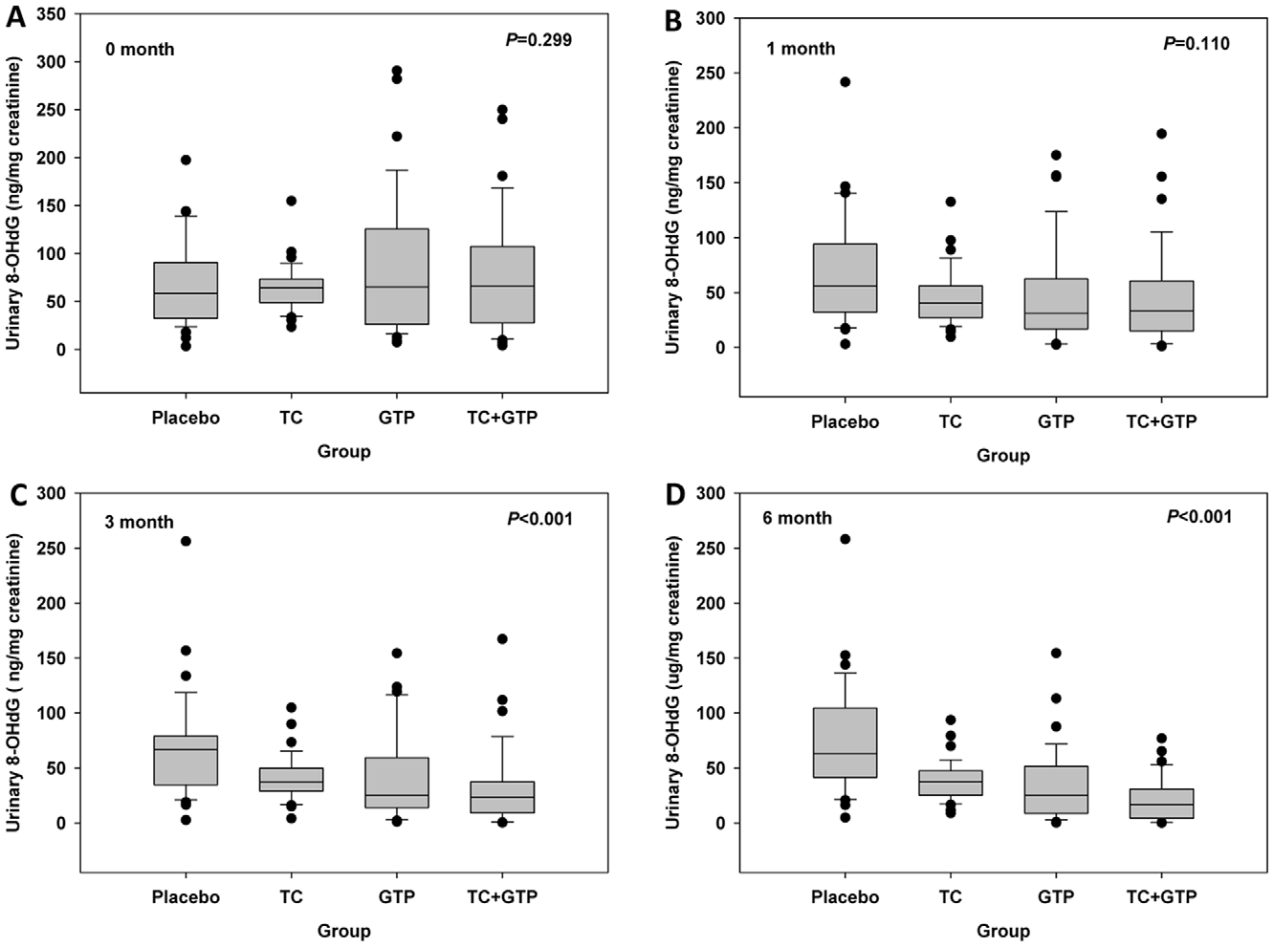
Urinary 8-OHdG concentrations in study participants in different groups. A: baseline, no significant differences in urinary 8-OHdG were found (*P* = 0.299); B: 1 month, urinary 8-OHdG concentrations were reduced by all treatments, but no significant differences were observed (*P* = 0.110); C: 3 month, urinary 8-OHdG concentrations were significantly reduced in all treatment groups (*P*<0.001); D: 6 month, urinary 8-OHdG concentrations were significantly reduced in all treatment groups (*P*<0.001).

**Figure 5:**
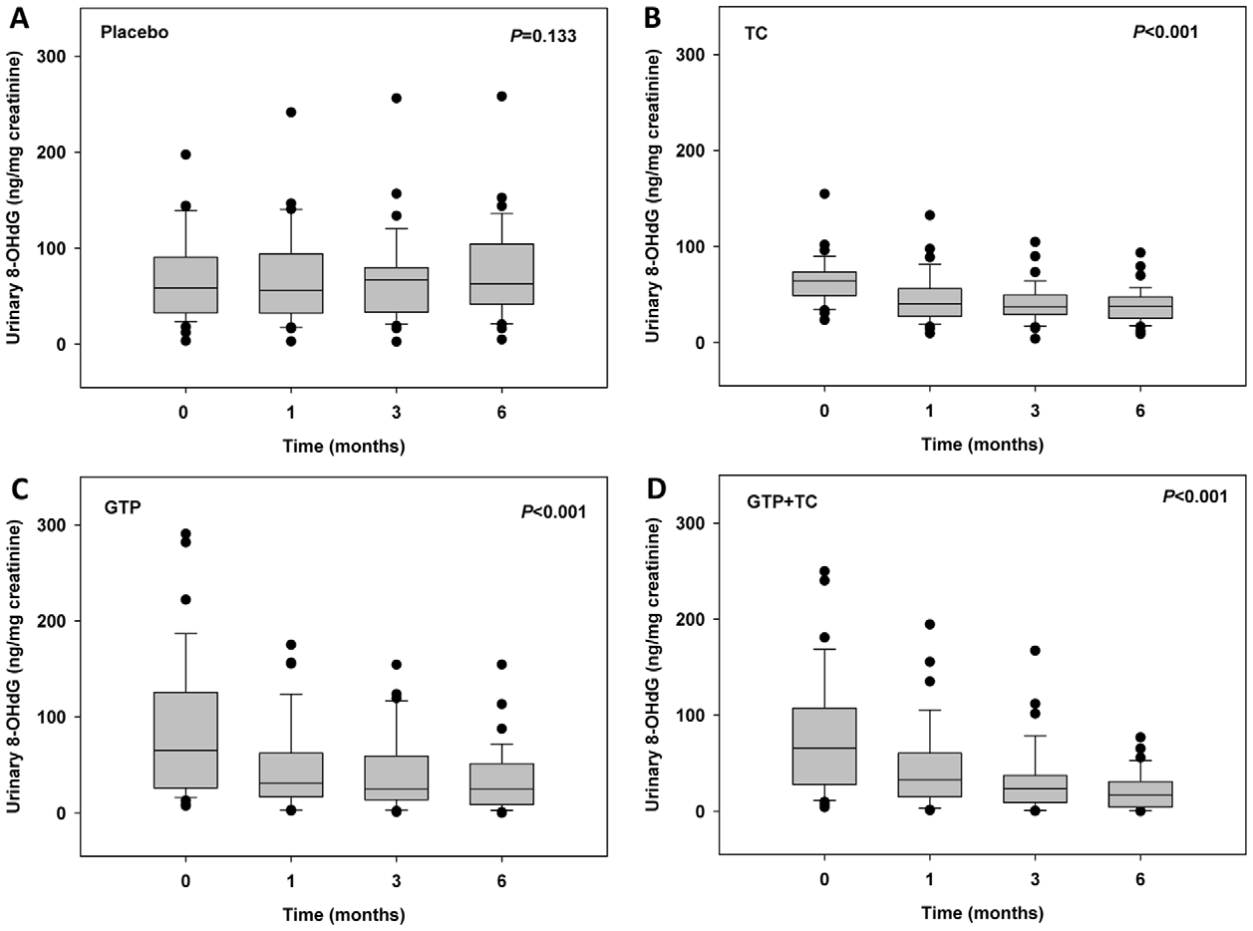
Temporal patterns of urinary 8-OHdG concentrations in study participants. A: Placebo, no significant differences was observed in different treatment periods (*P* = 0.133); B: TC, compared to baseline, urinary 8-OHdG concentrations were significantly reduced at 1-, 3-, and 6- months treatment (*P*<0.001); C: GTP, compared to baseline, urinary 8-OHdG concentrations were all significantly reduced at 1-, 3-, and 6-months treatment (*P*<0.001); and D: GTP+TC, compared to baseline, urinary 8-OHdG concentrations were all significantly reduced at 1-, 3- and 6-months treatment (*P*<0.001).

Urinary concentrations of 8-OHdG in study participants from each treatment group at the baseline, 1-, 3-, and 6-months of the intervention trial were summarized in Table 1, Figures 4 and 5. Data in Table 1 included the mean concentration of 8-OHdG with standard deviation, median, and detectable range. Detailed distribution of the whole dataset was shown in Figures 4 and 5. As shown in Figure 4A, the mean baseline concentrations among 4 treatment groups, although varied, were comparable (*P* = 0.299). After 1-month intervention, though no statistical significance was found among groups, the median concentration of 8-OHdG decreased by 27.4%, 43.9%, and 40.1% in TC, GTP, and GTP+TC treated groups, respectively (Figure 4B). After 3-months intervention, the median concentration of 8-OHdG decreased by 44.1%, 62.4%, and 64.8% in TC, GTP, and GTP+TC treated groups, respectively (Figure 4C), which showed statistical significance among treatment groups (*P*<0.001). After 6-months intervention, the median concentration of 8-OHdG decreased by 40.1%, 60.1%, and 73.3% in TC, GTP, and GTP+TC treated groups, respectively (Figure 4D), which also showed statistical significance among treatment groups (*P*<0.001).

**Table 1:** Urinary 8-OHdG concentrations in study participants in the intervention trial.

		Urinary 8-OHdG (ng/mg creatinine)				
Groups	No. of participants	Baseline	1-month	3-month	6-month	*P*-values (Time)^2^
Placebo	n = 37	64.5±42.7	65.8±48.5	68.3±48.1	73.5±51.0	0.133
		58.0 (3.5–197.4)	55.4 (3.0–241.6)	66.5 (2.6–256.2)	62.6 (4.9–258.2)	
Placebo + TC	n = 37	64.1±23.6	45.6±25.4^3,4^	40.6±20.2^3,4^	37.8±17.7^3,4^	<0.001
		65.1 (23.5–154.9)	40.2 (9.6–132.6)	37.2 (4.0–104.8)	37.5 (8.9–93.6)	
GTP	n = 39	84.6±74.3	48.2±44.6^3,4^	38.7±37.8^3,4^	33.5±33.1^3,4^	<0.001
		65.4 (7.6–290.6)	31.1 (2.5–175.1)	25.0 (1.1–154.4)	25.0 (0.5–164.5)	
GTP + TC	n = 37	75.8±61.0	45.7±43.5^3,4^	32.8±34.7^3,4^	20.7±19.8^3,4^	<0.001
		65.9 (4.2–249.8)	33.2 (1.2–194.5)	23.4 (0.5–167.2)	16.7 (0.5–76.8)	
*P*-values (Treatment)^1^		0.299	0.110	<0.001	<0.001	

Urinary 8-OHdG values are expressed as Mean±SD, median (range).

1 *P*-values (Treatment) were from Kruskal-Wallis test of one-way ANOVA.

2 *P*-values (Time) were from Friedman’s test.

3 Significantly different from Placebo at the same time point, tested by Wilcoxon rank sum test with Bonferroni correction for multiple comparisons, *P*<0.050.

4 Significantly different from baseline in the same group, tested by Wilcoxon signed rank test with Bonferroni correction for multiple comparisons, *P*<0.001.

Over the 6-month period, urinary 8-OHdG concentrations in the placebo group showed minimal changes as compared with the baseline level and no significant time effect was found (*P* = 0.133, Figure 5A). As shown in Figure 5B, urinary 8-OHdG concentrations in TC group significantly decreased over intervention time (*P*<0.001). The time effect was even more striking in GTP group (*P*<0.001, Figure 5C) and GTP+TC group (*P*<0.001, Figure 5D), suggesting that treatment with TC, GTP, or GTP+TC significantly decreased 8-OHdG concentrations in study participants.

### 5. Non-parametric Model Analysis of Treatment and Time on Concentrations of Urinary 8-OHdG

The effects of treatment and time interactions on urinary 8-OHdG concentrations were analyzed in a non-parametric model for repeated measures, which were summarized in Table 2. Statistical significances in the main effect of treatment and time and their interaction were found by this model analysis (*P*≤0.001). The treatment of TC, GTP and GTP+TC all had significant effects on urinary 8-OHdG concentrations, as compared with the placebo group (*P*<0.050); the urinary concentrations of 8-OHdG were also significantly modified by time for each of the treatment group (*P*<0.050). For each group, the duration of treatment proved to have cumulative effects on urinary 8-OHdG in the six month period. These results demonstrated the efficacy of treatment and time on lowering urinary 8-OHdG concentrations in study participants in this trial.

**Table 2:** Non-paramatric model analysis of treatment and time on concentrations of urinary 8-OHdG.

Effect	Test statistic (*P*-value)
Treatment	5.58 (*P* = 0.001)
Time	97.92 (*P*<0.001)
Treatment*Time	17.15 (*P*<0.001)
TC v.s. Placebo	10.62 (*P* = 0.001)
GTP v.s. Placebo	3.76 (*P* = 0.037)
(GTP+TC) v.s. Placebo	4.80 (*P* = 0.029)
TC*Time (time effect in TC group)	13.38 (*P*<0.001)
GTP*Time (time effect in GTP group)	67.20 (*P*<0.001)
(GTP+TC)*Time(time effect in (GTP+TC) group)	3.24 (*P* = 0.043)

The test statistics and *P*-values shown here were generated from a non-parametric model designed for repeated measurements using SAS macro F1_LD_F1, the more detailed information on model assumption and hypothesis is referred to reference #30, chapter 8.

## Discussion

In this study, we assessed the adherence and compliance of the 6-month clinical trial by measuring GTP components in blood and urine of the participants. Our previous studies have validated that measurement of blood and urinary GTP components are good biomarkers for determining green tea consumption and GTP intervention in human population studies [15], [27]. Among the four major GTP components, all EGCG, EGC, EC, and ECG, were detectable in blood and urine samples [16], but only EGCG and ECG in serum and EC and EGC in urine showed dose-response correlations [27]. Results of serum concentrations of EGCG and ECG and urinary excretion of EC and EGC in this study showed significant elevation after GTP intervention and no detectable concentrations in the baseline and the placebo participants over the study period, which further confirmed the excellent adherence and compliance of the overall trial. In addition, these results were consistent with our previous findings, i.e., blood and urinary GTP components are practical and reliable biomarkers for green tea consumption [27].

In this study, we further assessed the efficacy of GTP supplement and TC exercise for their individual and combined effects on the oxidative DNA damage biomarker, 8-OHdG, in postmenopausal women with osteopenia in the 6-month clinical trial. Results from this study showed that individual GTP supplement, TC exercise, or the combination significantly decreased urinary excretion of 8-OHdG after 1-, 3-, and 6-months intervention in postmenopausal women, as compared with the placebo group, which demonstrated the efficacy of this trial in reducing the oxidative stress biomarker levels.

Several lines of evidence have indicated the role of ROS in induction of osteoporosis [10], [14]: ROS are capable of influencing the generation and survival of osteoclasts, osteoblasts, and osteocytes; ROS-activated FoxOs in early mesenchymal progenitors also leads to decreased osteoblastogenesis through disruption of Wnt signaling pathway [10]; ROS also increases serum osteopontin and transforming growth factor-β levels in iron overloaded rats, suggesting osteoclast-mediated bone resorption through the receptor activator of nuclear factor-κB/RANK ligand (RANK/RANKL) mediated signaling pathway [30]. In postmenopausal women, the reduced estrogen level might be a key factor in boosting the ROS level because estrogen has been reported to be able to diminish production of ROS and stimulate the activity of glutathione reductase [31]. The ability of green tea and GTP as potent ROS scavengers has been well recognized as the basis for its biological activity [16], [32]. Previous studies by others and ours showed strong evidence of protective effects of GTP on the capacity of bone formation due to its anti-oxidative stress potentials [33], [34]. An animal study shows that urinary excretion of 8-OHdG is a reliable indicator of oxidative stress in postmenopausal rats [9]. More importantly, urinary 8-OHdG concentration has also been widely used as a DNA oxidative damage biomarker in many human studies [11], . In this study we observed a significant decrease in urinary 8-OHdG concentrations in postmenopausal women appearing at 1- to 6-months after the GTP intervention as compared to the placebo control group, which further suggests evidence of oxidative stress in the study participants. The result of this study is consistent with the previous finding in HBsAg carrier groups, which also showed statistically significant reduced urinary 8-OHdG after 3-months GTP intervention at 500 mg or 1,000 mg/day [27].

TC exercise has been characterized as a moderate intensity mind-body exercise, coupling muscular activity with an internally directed focus, producing a temporary self-contemplative mental state [36]. In this study, the TC exercise significantly decreased urinary 8-OHdG concentrations after 3-months intervention as compared with the control group. The effect of exercise on oxidative stress has been reviewed [37]. It was reported that high-intensity exercise increased oxidative stress biomarkers, including 8-OHdG and malondialdehyde (MDA)-modified low-density lipoprotein in humans, whereas moderate exercise tended to decrease both indices of oxidative stress [38]. A recent study has also demonstrated that TC exercise stimulated endogenous antioxidant enzymes (superoxide dismutase, SOD) and reduced oxidative damage markers (MDA) in middle-aged adults [23]. These reports support our observation that TC, a moderate exercise, reduced oxidative stress in postmenopausal women.

This is the first study to investigate the combined effects of GTP and TC on oxidative DNA damage in postmenopausal women with osteopenia. An additive effect of GTP supplementation and TC exercise on suppression of oxidative stress was found, as evidenced by a 40% reduction of urinary 8-OHdG in the TC group and 60% reduction in the GTP group alone and a 73% reduction was observed in the GTP+TC group. Results of this study further suggest that a combinative intervention strategy, including dietary supplementation and moderate exercise, may be more effective in promoting bone health in postmenopausal women with osteopenia.

The strength of our study was in its design as a randomized and placebo controlled clinical trial that could limit the biases usually introduced through the design and data acquisition. Limitations to this study included a relatively small sample size that might limit the statistical power, such as parameters after 1-month of intervention. A wide inter-individual variation found in concentration of biomarkers measured may be due to the individual variations in GTP metabolism enzymes, endogenous susceptibility factors, and/or DNA repair capacity; the large variation in urinary 8-OHdG levels (43.9±42.1 ng/mg creatinine) has also been reported previously in females [35], which supports this finding in our study. The stratified randomization and non-parametric analysis used could limit potential biases (randomization) and increase the power of detecting the differences (non-parametric model analysis). The intent to treat (ITT) analysis could benefit the comparison; however, considering the comparable compliance and adherence rates for the treatment groups, the effect of dropouts might be minimal. On the other hand, the exclusion of dropouts into statistical analysis may not severely affect the statistical power as an originally estimated 140 participants (34 participants for each group) would be sufficient for a power of about 0.90 for the outcome of urinary 8-OHdG level.

In conclusion, this study provides evidence that 500 mg GTP supplement, Tai Chi exercise, either alone or together, can effectively reduce an oxidative stress biomarker in postmenopausal women with osteopenia. Based on the putative role of oxidative stress in osteoporosis and the observed beneficial effects on bone health as published previously, these intervention strategies hold the potential as an alternative tool to benefit the bone health in postmenopausal women which deserves further long-term human studies.

## Supporting Information

Checklist S1 **CONSORT Checklist.**
(DOC)

Protocol S1 **Trial Protocol.**
(PDF)

Safety and clinical outcomes S1. (ZIP)
