# Supplementary material for: Mitigation of Oxidative Damage by Green Tea Polyphenols and Tai Chi Exercise in Postmenopausal Women with Osteopenia
Source: PLoS One. 2012 Oct 31;7(10):e48090. doi: 10.1371/journal.pone.0048090 (PMC3485260; doi:10.1371/journal.pone.0048090)
Supplement: Safety and clinical outcomes S1. — (ZIP) [file pone.0048090.s003.zip › 2012 Clinical Outcomes.pdf]

Osteoporos Int (2012) 23:1541–1552
DOI 10.1007/s00198-011-1731-x

ORIGINAL ARTICLE

Effect of green tea and Tai Chi on bone health in postmenopausal osteopenic women: a 6-month randomized placebo-controlled trial

C.-L. Shen · M.-C. Chyu · J. K. Yeh · Y. Zhang · B. C. Pence · C. K. Felton · J.-M. Brismée · B. H. Arjmandi · S. Doctolero · J.-S. Wang

Received: 19 April 2011 / Accepted: 28 June 2011 / Published online: 16 July 2011
© International Osteoporosis Foundation and National Osteoporosis Foundation 2011

Abstract
Summary Postmenopausal women with osteopenia received green tea polyphenols (GTP) supplement and/or Tai Chi exercise for 6 months. Bone turnover biomarkers, calcium metabolism, and muscle strength were measured. This study showed that GTP supplementation and Tai Chi exercise increased bone formation biomarkers and improved bone turnover rate. Tai Chi exercise increased serum parathyroid hormone. GTP supplementation, Tai Chi exercise, and the combination of the two all improved muscle strength in postmenopausal women with osteopenia.

Partial results were presented at the Annual Meeting of American Society for Bone and Mineral Research.

C.-L. Shen (✉) · M.-C. Chyu · B. C. Pence
Department of Pathology,
Texas Tech University Health Sciences Center,
BB 198, 3601 4th street,
Lubbock, TX 79430-9097, USA
e-mail: Leslie.Shen@ttuhsc.edu

C.-L. Shen
Department of Laboratory Science and Primary Care,
Texas Tech University Health Sciences Center,
Lubbock, TX, USA

C.-L. Shen · Y. Zhang
Laura W. Bush Institute for Women's Health,
Texas Tech University Health Sciences Center,
Lubbock, TX, USA

M.-C. Chyu
Healthcare Engineering,
Texas Tech University,
Lubbock, TX 79409, USA

M.-C. Chyu
Department of Health, Exercise, and Sport Sciences,
Texas Tech University,
Lubbock, TX 79409, USA

M.-C. Chyu
Department of Mechanical Engineering,
Texas Tech University,
Lubbock, TX 79409, USA

J. K. Yeh
Applied Bench Core Laboratory, Winthrop-University Hospital,
Mineola, NY 11501, USA

Y. Zhang
Department of Community and Family Medicine,
Texas Tech University,
Lubbock, TX 79409, USA

C. K. Felton
Department of Obstetrics and Gynecology, Texas Tech University,
Lubbock, TX 79409, USA

J.-M. Brismée
Rehabilitation Sciences, Texas Tech University,
Lubbock, TX 79409, USA

B. H. Arjmandi
Department of Nutrition, Food & Exercise Sciences,
Florida State University,
Tallahassee, FL 32303, USA

S. Doctolero
Clinical Research Center,
Texas Tech University Health Sciences Center,
Lubbock, TX 79430, USA

J.-S. Wang
Department of Environmental Health Science,
University of Georgia,
Athens, GA 30602, USA

Introduction This study evaluated the effect of GTP supplementation and Tai Chi (TC) exercise on serum markers of bone turnover (bone-specific alkaline phosphatase, BAP, and tartrate-resistant acid phosphatase, TRAP), calcium metabolism, and muscle strength in postmenopausal osteopenic women.

Methods One hundred and seventy-one postmenopausal osteopenic women were randomly assigned to four groups: (1) placebo (500 mg starch/day), (2) GTP (500 mg GTP/day), (3) placebo + TC (placebo plus TC training at 60 min/session, three sessions/week), and (4) GTP + TC (GTP plus TC training). Overnight fasting blood and urine samples were collected at baseline, 1, 3, and 6 months for biomarker analyses. Muscle strength was evaluated at baseline, 3, and 6 months. One hundred and fifty subjects completed the 6-month study.

Results Significant increases in BAP level due to GTP intake (at 1 month) and TC (at 3 months) were observed. Significant increases in the change of BAP/TRAP ratio due to GTP (at 3 months) and TC (at 6 months) were also observed. Significant main effect of TC on the elevation in serum parathyroid hormone level was observed at 1 and 3 months. At 6 months, muscle strength significantly improved due to GTP, TC, and GTP + TC interventions. Neither GTP nor TC affected serum TRAP, serum and urinary calcium, and inorganic phosphate.

Conclusion In summary, GTP supplementation and TC exercise increased BAP and improved BAP/TRAP ratio. TC exercise increased serum parathyroid hormone. GTP supplementation, TC exercise, and the combination of the two all improved muscle strength in postmenopausal women with osteopenia.

Keywords Bone turnover biomarker · Calcium metabolism · Green tea · Mind–body exercise · Muscle strength · Osteoporosis · Tai Chi

Introduction

Osteoporosis is a degenerative bone disease characterized by low bone mass and micro-structural deterioration of bone tissue leading to fragility and an increased risk for fractures, especially of the hip, spine, and wrist [1]. Postmenopausal women are four times more likely to develop osteoporosis than men because of a decrease in estrogen level after menopause causing decreased bone mineral density (BMD) and deteriorated bone microstructure [2].

Studies (both cross-sectional and retrospective) have shown that tea and its bioactive component may benefit bone health by maintaining BMD [3–8] and reducing the risk of fracture [9, 10]. Our animal studies [11, 12] have also shown that the osteo-protective effects of tea and its bioactive components may be mediated through enhanced osteoblastic activities and suppressed osteoclastic activities in bone remodeling. A positive correlation [4–7, 13–16], a weak inverse correlation [17], and no correlation [18–20] between tea drinking and BMD have been reported in published human studies. However, these results were based on cross-sectional or retrospective studies with inconsistent conclusion. Furthermore, no study has ever investigated the effect of tea and its bioactive components on bone biomarkers as well as bone metabolism in postmenopausal women.

Among different categories of tea, green tea seemed to benefit BMD more than others (e.g., white, black tea, and Oolong). Green tea polyphenols (GTP, extract of green tea) has shown its osteo-protective effects via decreasing oxidative stress [21], increasing activity of antioxidant enzymes [21], and decreasing expression of proinflammatory mediators [22] in various rodent bone loss models. In addition, green tea extract has been demonstrated to improve muscle function in mice [23], and combined tea catechin and treadmill exercise intervention mitigates aging-related degeneration in physical performance of mice [24]. However, limited information is available on the protective effect of consumption of tea or its bioactive components (i.e., GTP) on musculoskeletal health in postmenopausal women.

Tai Chi (TC) exercise, a weight-bearing mind–body exercise with aerobic and muscular fitness activity, has shown its potential to benefit musculoskeletal health [25–31] in terms of decelerating bone loss [29], preserving BMD [30] or improving neuromuscular function [31]. Among the previous studies, there was only one longitudinal study [29], but that study did not target a high-risk population with osteopenia or osteoporosis. Besides, that study employed a complicated 108-form TC which was difficult to learn for the US population [29]. A cross-sectional study failed to identify the types of regular TC exercise practiced by the women included in the study [30].

We have reported that GTP supplementation increased bone formation and reduced bone resorption in aged ovariectomized rats, an animal model of postmenopausal osteoporosis [11, 21]. In another study, we found that 6 weeks of TC exercise induced an increase in serum bone formation biomarker (bone-specific alkaline phosphatase, BAP) in healthy elderly participants [27]. Further, postmenopausal women with lower lean muscle mass or strength are at a higher risk for osteoporosis [32–36], while GTP [37–41] and TC [42, 43] may improve muscle strength. Based on these findings, the objective of the present study is to evaluate the effect of GTP supplementation and TC exercise on serum markers of bone turnover, calcium metabolism, and muscle strength in postmenopaus-

al osteopenic women. The hypothesis is that GTP intake and TC exercise alone and in combination would increase bone formation, decrease bone resorption, improve calcium metabolism, and improve muscle strength in postmenopausal women with low bone mass.

Methods

Study design and sample size calculation

This was a 6-month, placebo-controlled and randomized intervention trial to investigate the effects of GTP and TC on relevant primary and secondary outcomes in postmenopausal women with osteopenia. The primary outcome measures included a bone formation biomarker (bone-specific alkaline phosphatase, BAP), and a bone resorption biomarker (tartrate-resistant acid phosphatase, TRAP), while the secondary outcome parameters included calcium metabolism parameters such as serum parathyroid hormone (PTH), serum and urinary calcium (Ca), inorganic phosphate (Pi), creatinine, and muscle strength assessments.

The minimum sample size for assessing primary outcome measures was calculated based on data of bone biomarkers from previous studies [27, 44]. The baseline measurements values for control (placebo) and intervention groups (GTP, Placebo + TC, GTP + TC) were assumed the same as that in the previous study [27]. Intervention groups were expected to exhibit certain percentage changes in outcome measures in the follow-up visits, while the placebo group was expected to exhibit no changes throughout the study. It was further assumed that the correlation between baseline and the follow-up measurements is 0.85 and the correlation between the follow-up measurements is 0.90. Power analysis showed that a sample size yielding a power of approximately 0.85 to 0.9 at $\alpha=0.05$ for detecting differences in primary outcome measures was 120 participants. With an expected attrition rate of 15% over 24 weeks of intervention, a minimal sample size of 140 participants was needed.

Participants

Postmenopausal women were recruited primarily through flyers, local TV, radios, newspaper, municipal community centers, and clinics to participate in this study. The complete study protocol [45] has been published and only a brief description is provided here.

Of 315 potential participant candidates who showed interest and willingness of participation, 171 were recruited after screening based on the inclusion and exclusion criteria. The inclusion criteria were (1) postmenopausal women (at least 2 years after menopause) with osteopenia (mean lumbar spine and/or hip BMD T-score between 1 and 2.5 standard deviation (SD) below the young normal sex-matched areal BMD of the reference database); (2) normal function of thyroid, liver, and kidney; (3) serum alkaline phosphatase, Ca, and Pi within normal ranges; and (4) serum 25-hydroxy-vitamin D [25(OH)D] ≥20 ng/mL. Women were excluded if they (1) had a disease condition or were on medication known to affect bone metabolism; (2) had a history of cancer except for treated superficial basal or squamous cell carcinoma of the skin; (3) had uncontrolled intercurrent illness or physical condition that would be a contraindication to exercise; (4) had depression, cognitive impairment; or (5) were unwilling to accept randomization. Written informed consent was obtained from all the participants before enrollment. The study was approved by the Texas Tech University Health Sciences Center Institutional Review Board.

Randomization and blinding

To ensure comparable distribution across treatment arms, 171 participants were stratified by a fixed randomization scheme based on age (≥65 or <65 years old), habit of green tea consumption (yes or no), or current usage of estrogen/hormone replacement treatment (yes or no), and randomly assigned into placebo, GTP, Placebo + TC, or GTP + TC group.

Both the study participants and investigators responsible for the day-to-day operation and data analyses were blinded to the placebo or GTP group status. The identity of each treatment was revealed to the investigators and research personnel involved in the collection and analyses of the data only after all analyses have been completed.

Study intervention

Participants in the placebo and the placebo + TC groups received two capsules containing a total of 500 mg medicinal starch daily (one capsule ingested after breakfast and the other after dinner). Participants in the GTP and the GTP + TC groups received two capsules containing a total of 500 mg of GTP daily (one capsule ingested after breakfast and the other after dinner). Medicinal starch and GTP study capsules were supplied by Zhejiang Yuxin Pharmaceutical Co., Ltd., China (US FDA IND number 77,470). Based on our laboratory analysis, the GTP was 99.25% pure, with 46.5% of epigallocatechin-3-gallate (EGCG), 21.25% of epigallocatechin (ECG), 10% of epicatechin (EC), 7.5% of epicatechin-3-gallate (EGC), 9.5% of gallocatechin gallate (GCG), and 4.5% of catechin. In addition, during the 6-month intervention, all participants were supplemented with 500 mg elemental calcium and 200 IU vitamin D (as cholecalciferol) daily.

Participants who are randomized into the TC groups (Placebo + TC and GTP + TC) were expected to attend three 1-h TC exercise group classes each week for 6 months. In each session, in addition to 10 min of warm-up and 5 min of cool-down exercises, the routine of 24-form simplified Yang-style TC was repeated six times during the 45-min training period based on the standard speed of about 7 min per routine [46]. The 24-form simplified Yang-style TC is a TC routine for beginners based on the traditional Yang-style TC long form. It consists of 24 movements of martial application performed slowly and gently while breathing deeply and meditating. The instructor explained and demonstrated how the exercise should be performed, and subjects followed. Participants in non-TC groups (Placebo and GTP groups) were asked to continue their customary activity levels throughout the study period.

Data collection

Data of medical history, physical activity level, depression (mood), and cognitive function were collected at the time of enrollment. Physical activity level was assessed with Godin Leisure-Time Exercise Questionnaire [47]. Depression level was assessed with Yesavage self-rated Geriatric Depression Score [48]. Cognitive function was assessed with Mini-Mental State Examination [49].

BMD was measured at baseline for screening purpose by dual energy X-ray absorptiometry (DEXA) (Norland Excel X-Ray Bone Densitometer). At baseline, 1, 3, and 6 months, overnight fasting blood and urine samples were collected. All outcomes were measured at baseline, 1, 3, and 6 months, except for muscle strength assessment (measured at baseline, 3, and 6 months). In addition, at 3 and 6 months, physical activity level was monitored for any deviation from routine physical activity level throughout the study period.

Compliance and adverse event monitoring

Compliance of GTP or placebo study agents was determined as the amount of capsules ingested divided by the amount a participant should have ingested throughout the study period. Adherence of TC classes was assessed by TC classes attended divided by all those a participant should have attended. In the course of the study, adverse events associated with study agents or TC exercise were self-reported by the participants. In addition, the activities of aspartate aminotransferase (AST) and alanine aminotransferase (ALT) in serum were determined monthly in order to monitor liver function.

Measurement of bone turnover biomarkers and blood chemistry

Serum BAP levels were measured by enzyme-linked immunoassay (Quidel Corporation, San Diego, CA, USA) with intra- and inter-assay CV below 5.2% and 5.0%, respectively. Serum TRAP levels were measured by immunocapture enzyme assay (Quidel Corporation) with intra- and inter-assay CV below 2.2% and 3.0%, respectively. Laboratory blood and urinary chemistry parameters, including serum 25(OH)-D, thyroid stimulating hormone (TSH), intact PTH, AST, ALT, Ca, Pi, and creatinine as well as urinary Ca, Pi, and creatinine were analyzed by a certified diagnostic laboratory (Quest Diagnostic Laboratory, Dallas, TX, USA).

Assessment of static leg strength/endurance

The wall-sit test, also referred to as the wall-slide test, was employed to assess static leg strength and endurance, particularly of the quadriceps and hip extensors [50, 51]. A subject first stood in front of a bench (12″ high) positioned against a vertical smooth wall, with her back facing the wall and feet shoulder-width apart. She then leaned back so that her back and buttock were against the wall. With her hands on the waist, the subject flexed her knees, lowering her torso down the wall to assume a position with both her hips and knees flexed at a 90° angle (measured with a goniometer) without touching the bench. The longest time (seconds) that the subject can continuously hold this position before sitting down on the bench was recorded [52].

Statistical analysis

All variables were expressed as mean±standard deviations (SD) or standard error of mean (SEM), unless otherwise indicated. Statistical significance was set at the level of $p < 0.05$. Statistical analyses of data were performed using SPSS version 17.0 (SPSS Inc., Chicago, IL, USA). Differences in baseline characteristics including demographic information (e.g., age and general health) among the groups were evaluated by one-way ANOVA. The percent changes in serum BAP/TRAP ratio and in serum PTH were calculated [(post intervention value−baseline value)/baseline value×100%] for each group. The group membership (Placebo, GTP, Placebo + TC, or GTP + TC) is the main independent variable (i.e., the factor). One-way ANCOVAs were performed to determine the main effects of GTP intake, TC exercise, and their interaction on the primary and secondary outcomes at baseline, 1, 3, and 6 months. *Post*

hoc comparisons (Bonferroni adjustment) were conducted to compare the individual outcome difference between the four groups when ANCOVA showed significance. A mixed model of ANOVA (i.e., a repeated measures model that also includes a factor variable in SPSS) was performed to examine impact of the intervention, time, and their interaction on the primary and secondary outcomes. The between-subject factor is the group membership (i.e., type of intervention received), and the within-subject factor is time (three time points of measurement).

Results

Study participants

Figure 1 shows the study flow chart with details of the participants. A total of 171 subjects were recruited and 150 subjects completed the study. Seven (16%) participants in the Placebo arm, eight (17%) in the GTP arm, five (12%) in the Placebo + TC arm, and one (3%) in the GTP + TC arm withdrew before the end of the study due to accidental fall at home (one subject), relocation (two subjects), time conflicts (six subjects), lost to follow-up (five subjects), and lost interest (seven subjects). Baseline characteristics were similar among all treatment groups (Table 1). No statistically significant differences between the subjects who withdrew from the study and those who completed the study were observed in any parameter. Based on the results of pill count, the compliance rate was 89% for both GTP and placebo capsules. The adherence rate for TC classes was 83%. Neither GTP supplementation nor TC exercise affected serum ALT and AST activity (indicators of liver function) throughout the study (data not shown). No adverse event due to study treatment was reported by the participants.

Overall, mix-ANOVA model did not find a significant impact of the intervention when intervention, time, and their interaction were included in one model. However, separate one-way and two-way ANCOVA revealed some significant findings at different time points.

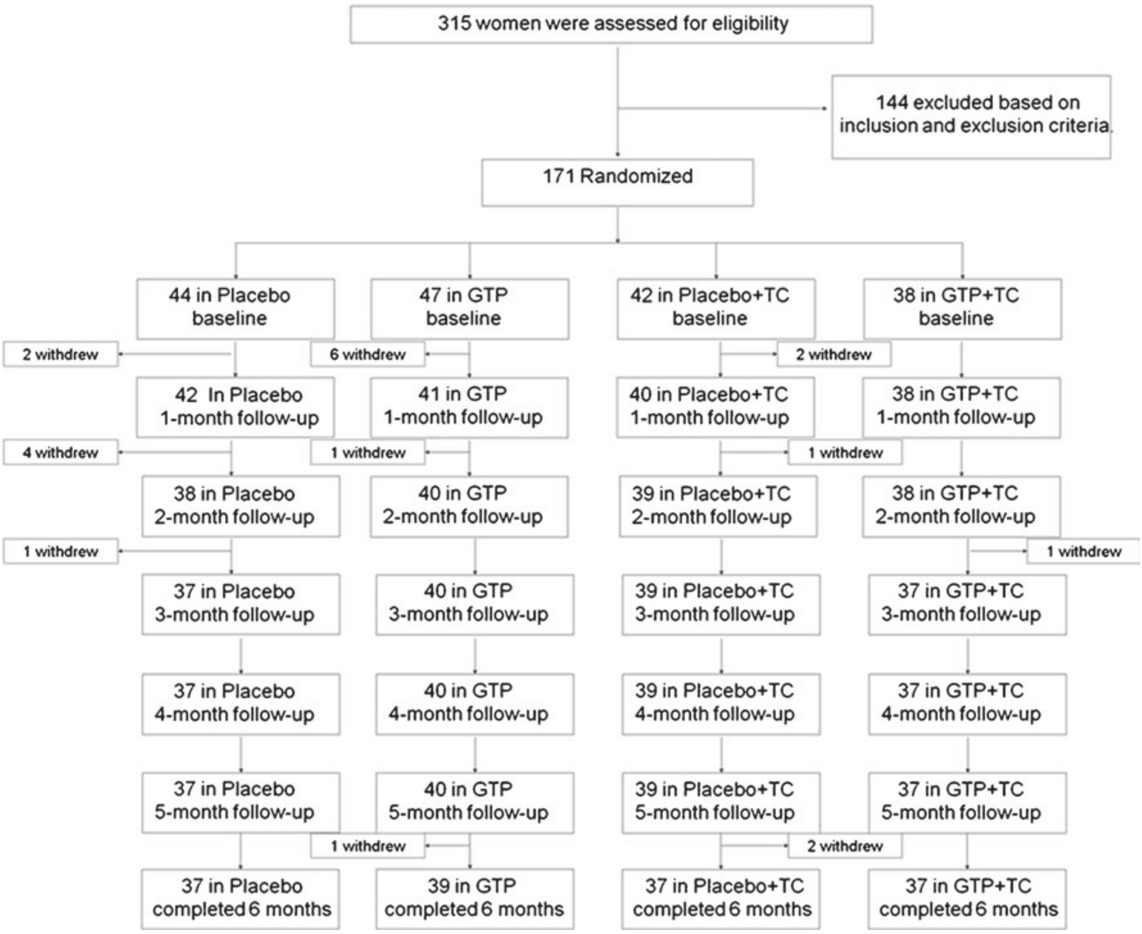

Fig. 1 Study flow chart

Table 1 Baseline demographic characteristics of study population

Variables	Placebo	GTP	Placebo + TC	GTP + TC	*p* values
Number	44	47	42	38	
Age (years)	57.6±7.5	56.5±5.5	58.3±7.7	57.6±6.7	NS
Years after menopause	12.5±8.4	11.2±8.3	14.2±11.1	11.4±9.1	NS
Height (cm)	162.8±7.8	162.7±5.4	161.9±6.2	164.1±7.1	NS
Weight (kg)	77.4±15.4	74.5±11.9	74.1±12.1	72.9±14.6	NS
Body mass index (kg/m^2)	29.2±6.1	28.1±4.4	28.3±4.9	27.1±5.8	NS
Bone mineral density (T-score)					
Femoral neck	−1.50±0.62	−1.51±0.64	−1.64±0.56	−1.69±0.59	NS
Trochanter	−1.05±0.81	−1.10±0.70	−1.27±0.62	−1.29±0.71	NS
Total spine	−0.74±0.56	−0.75±0.67	−0.95±0.65	−0.97±0.51	NS
L1–L4	−0.60±0.85	−0.53±1.09	−0.65±0.76	−0.75±0.83	NS
Serum 25(OH)D (ng/mL)	32.5±8.4	36.2±11.0	31.5±11.5	30.1±6.6	NS
Serum PTH (pg/mL)	47.8±20.1	45.6±22.5	48.2±22.3	47.2±27.1	NS
Serum TSH (mIU/L)	2.50±1.38	2.09±1.13	2.38±0.94	2.56±1.30	NS
General health questions (*n*, %)					
General health rated "good"	35 (79.5)	38 (80.9)	38 (90.4)	30 (78.9)	NS
Height decrease with age	3 (6.8)	10 (21.2)	7 (16.6)	9 (23.6)	NS
Broken bone as adult	9 (20.4)	13 (27.6)	7 (16.6)	14 (36.8)	NS
History of osteopenia	9 (20.4)	8 (17.0)	9 (21.4)	9 (23.9)	NS
Family history of low bone mass	19 (36.3)	24 (51.0)	13 (30.9)	23 (60.5)	NS
History of osteoarthritis	9 (20.4)	5 (10.6)	4 (9.5)	4 (10.5)	NS
Severe joint or muscle pain	16 (36.3)	12 (25.5)	9 (21.4)	9 (23.6)	NS
Back or leg pain	11 (25.0)	12 (25.5)	4 (9.5)	5 (13.1)	NS
History of diabetes	1 (2.3)	5 (10.6)	6 (14.2)	3 (7.8)	NS
History of hypertension	13 (29.5)	9 (19.1)	10 (23.8)	8 (21.0)	NS
Physical activity profiles					
Exercise frequency (sessions/week)	2.4±2.1	2.0±2.1	1.8±2.0	1.9±2.0	NS
Exercise time (min/session)	22±19	23±21	29±46	23±22	NS
Mood assessment[a]	5.7±5.2	5.9±4.9	5.9±5.6	6.3±4.2	NS

All data are mean ± standard deviation unless otherwise specified. *NS* no significant difference ($p>0.05$) between groups was observed in any item

GTP green tea polyphenols, *TC* Tai Chi, *25(OH)D* 25-hydroxy-vitamin D, *PTH* parathyroid hormone, *TSH* thyroid stimulating hormone, *HRT* hormone replacement treatment

[a] Mood assessment was performed by the Yesavage self-rated Geriatric Depression Score

Bone turnover biomarkers

Data of bone turnover biomarkers are exhibited in Table 2. One-way ANOVA revealed no significant difference among the four groups at baseline in terms of serum BAP and serum TRAP ($p>0.05$). Based on two-way ANCOVA results, significant main effects of GTP intake and TC exercise were found on serum BAP. GTP groups (GTP and GTP + TC) had higher serum BAP values at 1 month ($p=0.03$), while TC groups (Placebo + TC and GTP + TC) had higher BAP values at 3 months ($p=0.04$) (Table 2). However, neither GTP supplementation nor TC exercise had any effect on serum TRAP levels (bone resorption biomarker) at any time point ($p>0.05$). No changes in serum BAP and TRAP were observed due to TC and GTP at 6 months. No interaction effect between GTP supplementation and TC exercise was observed in serum BAP and TRAP at any time point.

Figure 2 displays the results of the changes in the BAP/TRAP ratio. Similar to the results for individual bone biomarkers, GTP groups (GTP and GTP + TC groups) demonstrated higher values for the percent change in BAP/TRAP ratio at 3 months compared to the non-GTP groups (Placebo and Placebo + TC groups) ($p=0.011$). TC exercise groups (Placebo + TC and GTP + TC groups) also demonstrated higher values for the percent change in BAP/TRAP ratio at 6 months compared to the non-TC

Table 2 Effects of 6-month GTP supplementation and TC exercise on bone turnover biomarkers in postmenopausal osteopenic women

Variables	Treatment groups			
	Placebo	GTP	Placebo + TC	GTP + TC
Serum BAP (bone formation biomarker), U/L				
Baseline	31.7±1.5	31.3±1.4	32.1±1.5	35.0±1.5
	(14.7–48.8)	(16.2–54.6)	(19.6–50.4)	(14.2–70.9)
1-month	31.1±1.6	33.0±1.5 [a]	31.7±1.6	36.6±1.6[a]
	(15.9–49.9)	(11.6–70.6)	(17.6–53.7)	(20.7–65.2)
3-month	30.5±1.6	32.0±1.5	32.7±1.6[b]	36.3±1.6[b]
	(13.3–49.6)	(12.9–58.4)	(17.8–68.8)	(20.0–64.7)
6-month	31.5±1.6	32.9±1.6	33.0±1.7	37.3±1.7
	(12.1–54.8)	(15.7–66.0)	(16.3–79.3)	(21.0–68.2)
Serum TRAP (bone resorption biomarker), U/L				
Baseline	3.49±0.19	3.15±0.18	3.43±0.19	3.49±0.19
	(1.26–7.15)	(1.75–5.46)	(1.70–7.41)	(1.73–6.83)
1-month	3.29±0.21	3.08±0.20	3.21±0.21	3.30±0.20
	(1.23–7.77)	(1.57–6.13)	(1.44–7.68)	(1.65–6.89)
3-month	3.21±0.20	2.93±0.19	3.32±0.20	3.13±0.20
	(1.16–7.50)	(1.74–5.50)	(1.28–9.84)	(1.47–5.96)
6-month	3.28±0.20	3.12±0.20	3.13±0.21	3.30±0.21
	(1.05–8.52)	(1.89–5.98)	(1.69–9.09)	(1.58–6.69)

Data are mean ± SEM. Range is also listed

GTP green tea polyphenols, *TC* Tai Chi, *BAP* bone-specific alkaline phosphatase, *TRAP* tartrate-resistant acid phosphatase

[a] Significant main effect of GTP intake on serum BAP at 1 month ($p=0.03$) shown by the two-factor ANCOVA model

[b] Significant main effect of TC exercise on serum BAP at 3 months ($p=0.04$) shown by the two-factor ANCOVA model

groups (Placebo and GTP groups) ($p=0.011$). No interaction effect between GTP and TC on the change of BAP/TRAP ratio was observed at any time point.

Calcium metabolism parameters

Table 3 presents the data of serum/urinary Ca, Pi, and creatinine. One-way ANOVA showed no significant difference among the four treatment groups in serum and urinary Ca and Pi at the baseline. Neither GTP nor TC affected serum/urinary calcium, inorganic phosphate, or creatinine. Figure 3 shows the percent change in serum intact PTH from baseline to 6 months. There was no significant difference among all four groups at the baseline ($p>0.05$). Two-way ANCOVA showed that TC exercise (Placebo + TC and GTP + TC groups) induced a significant elevation in the percent change in serum intact PTH levels at 1 month ($p=0.009$) and 3 months ($p=0.035$) than those in the non-TC groups (Placebo and GTP groups) (Fig. 3). No interaction was observed between GTP intake and TC exercise in the change in serum intact PTH levels.

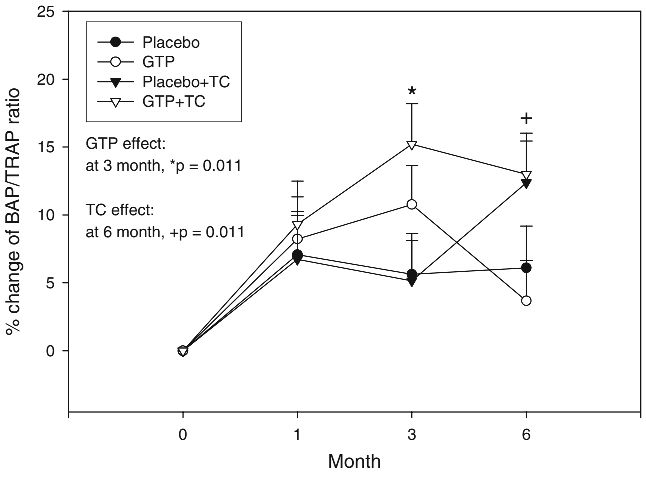

Fig. 2 Change of BAP/TRAP ratio (±SEM) relative to baseline. *Asterisk* indicates significant main effect of GTP intake on change in BAP/TRAP ratio after 3 months ($p<0.05$). *Plus sign* indicates significant main effect of TC exercise on change in BAP/TRAP ratio after 6 months ($p<0.05$)

Muscle strength

One-way ANOVA analysis revealed no difference in static leg strength/endurance measured by wall-sit test, at the baseline among the four treatment groups. Figure 4 exhibits the data of muscle strength during the 6-month study. Based on one-way ANOVA analysis, at 6 months, the Placebo group showed no statistically significant change in muscle strength, while all three treatments (GTP, Placebo + TC, and GTP + TC) significantly improved participants' static leg strength/endurance as shown by the longer wall-sit time duration ($p<0.001$ for the Placebo + TC group, $p<0.001$ for the GTP group, and $p=0.001$ for the GTP + TC group).

Table 3 Effects of 6-month GTP supplementation and TC exercise on serum and urinary Ca and Pi in postmenopausal osteopenic women

Variables	Treatment groups			
	Placebo	GTP	Placebo + TC	GTP + TC
Serum Ca, mg/dL				
Baseline	9.39±0.37	9.36±0.35	9.41±0.24	9.49±0.35
1-month	9.55±0.53	9.39±0.38	9.35±0.34	9.40±0.36
3-month	9.46±0.39	9.33±0.51	9.37±0.29	9.43±0.47
6-month	9.37±0.40	9.29±0.34	9.27±0.33	9.42±0.33
Serum Pi, mg/dL				
Baseline	3.70±0.47	3.67±0.60	3.67±0.49	3.74±0.40
1-month	3.87±0.58	3.62±0.52	3.66±0.45	3.65±0.48
3-month	3.97±0.87	3.77±0.50	3.81±0.48	3.75±0.41
6-month	3.70±0.43	3.62±0.61	3.76±0.48	3.74±0.43
Urinary Ca/Crt, mg/mg creatinine				
Baseline	97.8±66.8	86.1±76.6	97.7±64.7	97.7±68.9
1-month	109.2±70.1	85.6±72.4	104.2±71.7	102.7±62.2
3-month	118.0±96.8	76.1±46.1	123.3±92.9	100.7±63.9
6-month	118.8±72.6	90.9±71.3	118.5±80.8	118.1±68.9
Urinary Pi/Crt, mg/mg creatinine				
Baseline	468±162	469±220	500±206	504±243
1-month	450±185	449±233	478±217	467±212
3-month	480±231	500±211	515±233	522±170
6-month	429±202	424±195	468±208	477±204

Data are mean ± SD

GTP green tea polyphenols, *TC* Tai Chi, *Ca* calcium, *Pi* inorganic phosphorus, *Crt* creatinine

Discussion

The present study is the first to assess GTP supplementation and TC exercise for their individual and conjugate effects on bone turnover biomarkers, calcium metabolism, and muscle strength in postmenopausal women with osteopenia.

We observed elevated BAP level in GTP and TC groups, respectively, at earlier time points during the intervention. Higher values of BAP, an indicator of increased bone formation, were observed after 1 month in the GTP supplemented group. This finding appears to be consistent with our previous animal study that GTP supplementation enhanced bone formation activity in ovariectomized rats, a model of postmenopausal osteoporosis [21]. The ability of GTP to increase an index of bone formation demonstrated

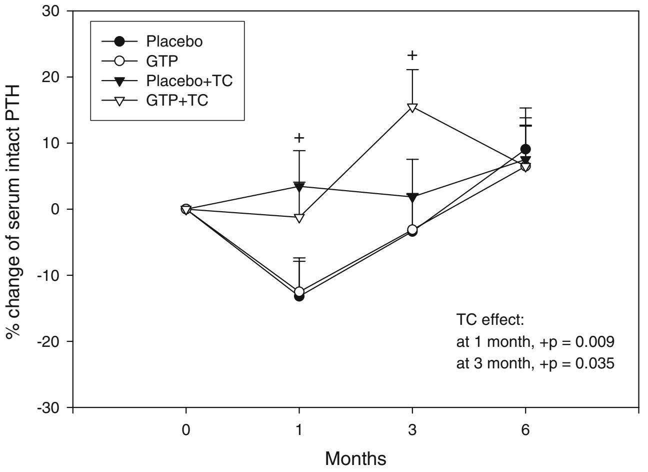

Fig. 3 Change of serum intact PTH (±SEM) relative to baseline. *Plus sign* indicates significant main effect of TC exercise on change in serum intact PTH at 1 and 3 months ($p<0.05$)

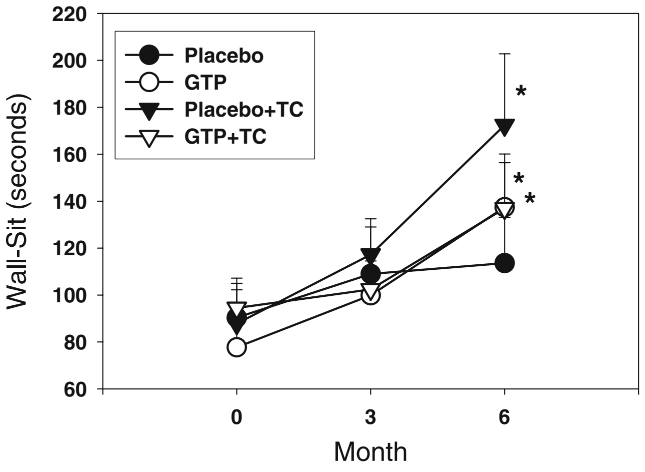

Fig. 4 Effects of GTP supplementation and TC exercise on muscle strength (static leg strength/endurance evaluated by wall-sit test) in postmenopausal osteopenic women. Data are mean±SEM. *Asterisk* indicates significant difference from baseline (month 0) for each treatment group ($p<0.001$ for the GTP group, $p<0.001$ for Placebo + TC group, and $p=0.001$ for GTP + TC group)

in our study also agrees with GTP's effect on osteoblastic activity suggested in in vitro studies [53–56].

In terms of impact of TC exercise on BAP, higher values of BAP observed after 3 months in the TC exercise groups agree with a previous study in an elderly population [27]. TC exercise involves shifting body weight so slowly that almost the entire body weight is supported alternately by one of the legs at all times during the exercise. As a result, a significant weight-bearing condition is created [27, 57] which may induce mechanical loading in bone, thus stimulating new bone formation. The disappearance of the effects of GTP and TC on BAP level after 6 months may be related to the bone adaptation to GTP or TC after a long intervention period.

In addition to individual bone biomarkers, the ratio of bone formation to resorption biomarkers (BAP/TRAP in this study) has been also used as an indicator of the state of bone turnover to evaluate the effect of exercises [58–60] and dietary supplements [61, 62] on bone metabolism. An increased BAP/TRAP ratio, which could result from an increase in bone formation or a decrease in bone resorption, indicates a state of bone turnover to increase bone formation due to the intervention [63]. On the other hand, a decreased BAP/TRAP ratio, which could result from a decrease in bone formation or an increase in bone resorption, indicates a state of bone turnover to increase bone resorption caused by the intervention [63]. In this study, the increased BAP/TRAP ratio at 3 and 6 months by GTP intake and TC exercise, respectively, are due to increased bone formation rather than decreased bone resorption (see Table 2). Therefore, such an increase in the BAP/TRAP ratio suggests that GTP intake and TC exercise increased bone formation.

In this study, we also noticed that all treatment groups, including placebo, demonstrated an increase in BAP/TRAP ratio during the study period. In the present study, all participating subjects were provided with calcium plus vitamin D supplement daily throughout the study period, as described in section "Study intervention". According to the reported data, the average calcium intake for our target population was about 700–800 mg daily [64, 65]. After taking the 500 mg of calcium provided by the study, our subjects would have taken 1,200–1,300 mg of calcium daily, compared to the recommended dietary allowance (RDA) (1,200 mg). Similarly, the vitamin D supplementation we provided was to ensure that all subjects had received the total RDA of vitamin D after that received from diet and sun exposure. Adequate calcium plus vitamin D intake is essential for bone activities in order to study the effect of dietary supplements and exercise on bone health [66, 67]. The increase of the BAP/TRAP ratio in the placebo group may be due to the supplementation of calcium plus vitamin D during the study.

PTH plays a key role in the changes of bone markers. PTH has been shown to play dual roles in the skeleton, anabolic and catabolic, in terms of stimulation of osteoblast and osteoclast differentiation, number, and activity [68]. Continuous administration of PTH can induce bone loss, where intermittent administration of PTH has been shown to increase cancellous bone volume [69] probably due to a stimulatory effect of local growth factors, such as insulin-like growth factors and transforming growth factor-beta [70]. In the present study, the findings that higher serum PTH level at TC exercise group was observed at 1 and 3 months in postmenopausal osteopenic women agrees with previous studies that different exercise forms including TC were related to raised serum PTH level [27, 57, 71, 72]. We also noticed that after 6 months, there was no difference in the change of PTH relative to baseline among four treatment groups, possibly because the bone cells may have adapted to stimuli (GTP intake or TC exercise in the present study) after a long period of time. It is not clear why TC exercise resulted in a higher serum PTH at 1 and 3 months and whether and how such transient increases in serum PTH would affect bone quality. These issues may need to be investigated in future studies.

Loss of estrogen and physical inactivity has been considered important contributors to loss of muscle mass and strength in postmenopausal women [73]. Recent studies suggested that postmenopausal women with lower lean muscle mass or strength at legs and trunk would be at a higher risk for osteoporosis and should be a target for preventive measures [32–36]. The present results showed that after 6 months of GTP supplementation, TC exercise, or their combined intervention, the static leg muscle strength/endurance in postmenopausal osteopenic women significantly improved. The positive impacts of green tea on muscle have been reported in rodent models [37–41]. Green tea extract has been shown to improve muscle health by reducing or delaying muscle necrosis in *mdx* mice, a model of muscular dystrophy [37–39]. Recent studies showed that combined tea catechin and exercise intervention palliates aging-related degeneration in physical performance and energy metabolism by improving mitochondrial function in skeletal muscle in mice [27] and enhances endurance capacity in maturing *mdx* mice possibly due to increased skeletal muscle lipid oxidation by green tea extract along with exercise [41]. Further, our findings that TC exercise improved muscle strength is supported by other studies reporting that regular TC practitioners had better muscle strength and endurance capacity than sedentary controls [42, 43], possibly because TC's movements of the lower limbs put more demand on ankle dorsiflexors [74], knee extensors [75], and knee muscle activation [76]. Therefore, TC exercise may provide bone protection by improving neuromuscular function in postmenopausal women [31].

We recognize the following limitations in the present study: (1) The number of subjects in each group was not equal. (2) The amount of dietary calcium intake for each subject was not available. However, there may be an interaction between calcium and treatments (GTP and/or TC). (3) Bone remodeling is a slow process, and the time required to complete a bone remodeling cycle may increase with age. Therefore, BMD could not be justified as an outcome in the present short-term study where changes in BMD were not expected to be detectable, although BMD is considered more closely related to clinical implication of the treatments than the biomarkers measured in this study. In addition, we have noticed that in the present study, the effects of GTP and TC on bone biomarkers were transient and none of the changes were significant at the 6-month time point. A long-term study is therefore recommended that measures BMD as the primary outcome using DEXA. In addition, evaluation of bone microstructure including volumetric BMD, stress–strain index, and bone geometry using advanced peripheral quantitative computed tomography (pQCT) can further elucidate the clinical impact of GTP supplementation, TC exercise, and their combined treatment.

Conclusion

The results of this study showed that both GTP supplementation and TC exercise increased bone formation biomarkers and improved bone turnover rate. TC exercise increased serum parathyroid hormone levels. GTP supplementation, TC exercise, and the combination of the two all improved muscle strength in postmenopausal women with osteopenia.

Acknowledgments This study was supported by the National Center for Complementary and Alternative Medicine (NCCAM) of the National Institutes of Health under grant 1R21AT003735-01A1. The contents of this manuscript are solely the responsibility of the authors and do not necessarily represent the official views of the NCCAM or the National Institutes of Health. We gratefully acknowledge the study participants; without them, this study would not have been possible. We would like to thank Dr. Jay Magaziner (University of Maryland, MD) for their advice. We also thank Mary J. Flores, Raul Y. Dagda, and Marisela Dagda for their assistance in data collection. Clinical trial number: NCT00625391.

Conflicts of interest None.

References

1. NIH (2001) Consensus Development Panel on Osteoporosis Prevention, Diagnosis, and Therapy. Osteoporosis prevention, diagnosis, and therapy. JAMA 285(6):785–795
2. Looker AC, Melton LJ 3rd, Harris TB, Borrud LG, Shepherd JA (2010) Prevalence and trends in low femur bone density among older US adults: NHANES 2005–2006 compared with NHANES III. J Bone Miner Res 25(1):64–71
3. Hamdi Kara I, Aydin S, Gemalmaz A, Aktürk Z, Yaman H, Bozdemir N, Kurdak H, Sitmapinar K, Devran Sencar I, Basak O, Akdeniz M, Isildar H, Burgut E, Ozcan S, Akca U, Dağdeviren N, Ungagn M (2007) Habitual tea drinking and bone mineral density in postmenopausal Turkish women: investigation of prevalence of postmenopausal osteoporosis in Turkey (IPPOT Study). Int J Vitam Nutr Res 77(6):389–397, Erratum in: Int J Vitam Nutr Res 2008;78(3):following 166
4. Muraki S, Yamamoto S, Ishibashi H, Oka H, Yoshimura N, Kawaguchi H, Nakamura K (2007) Diet and lifestyle associated with increased bone mineral density: cross-sectional study of Japanese elderly women at an osteoporosis outpatient clinic. J Orthop Sci 12(4):317–320
5. Hegarty VM, May HM, Khaw KT (2000) Tea drinking and bone mineral density in older women. Am J Clin Nutr 71(4):1003–1007
6. Hoover PA, Webber CE, Beaumont LF, Blake JM (1996) Postmenopausal bone mineral density: relationship to calcium intake, calcium absorption, residual estrogen, body composition, and physical activity. Can J Physiol Pharmacol 74(8):911–917
7. Devine A, Hodgson JM, Dick IM, Prince RL (2007) Tea drinking is associated with benefits on bone density in older women. Am J Clin Nutr 86(4):1243–1247
8. Wu CH, Yang YC, Yao WJ, Lu FH, Wu JS, Chang CJ (2002) Epidemiological evidence of increased bone mineral density in habitual tea drinkers. Arch Intern Med 162:1001–1006
9. Johnell O, Gullberg B, Kanis JA, Allander E, Elffors L, Dequeker J, Dilsen G, Gennari C, Lopes Vaz A, Lyritis G et al (1995) Risk factors for hip fracture in European women: the MEDOS Study. Mediterranean Osteoporosis Study. J Bone Miner Res 10:1802–1815
10. Kanis J, Johnell O, Gullberg B, Allander E, Elffors L, Ranstam J, Dequeker J, Dilsen G, Gennari C, Vaz AL, Lyritis G, Mazzuoli G, Miravet L, Passeri M, Perez Cano R, Rapado A, Ribot C (1999) Risk factors for hip fracture in men from southern Europe: the MEDOS Study. Mediterranean Osteoporosis Study. Osteoporos Int 9:45–54
11. Shen CL, Yeh JK, Stoecker BJ, Chyu MC, Wang JS (2009) Green tea polyphenols mitigate deterioration of bone microarchitecture in middle-aged female rats. Bone 44:684–690
12. Shen CL, Yeh JK, Samathanam C, Cao JJ, Stoecker BJ, Dagda RY, Chyu MC, Dunn DM, Wang JS (2011) Green tea polyphenols attenuate deterioration of bone microarchitecture in female rats with systemic chronic inflammation. Osteoporos Int 22:327–337
13. Chen X, Pettinger MB, Ritenbaugh C, LaCroix AZ, Robbins J, Caan BJ, Barad DH, Hakim IA (2003) Habitual tea consumption and risk of osteoporosis: a Prospective Study in the Women's Health Initiative Observational Cohort. Am J Epidemiol 158:772–781
14. Vestergaard P, Hermann AP, Gram J, Jensen LB, Eiken P, Abrahamsen B, Brot C, Kolthoff N, Sørensen OH, Beck Nielsen H, Pors Nielsen S, Charles P, Mosekilde L (2001) Evaluation of methods for prediction of bone mineral density by clinical and biochemical variables in perimenopausal women. Maturitas 40(3):211–220
15. Hong X, Lü H, Yang J, Li Z (2001) An analysis on the forearm bone mass density of rural female and the environmental risk factors. Wei Sheng Yan Jiu 30(4):227–230
16. Hernandez ER, Seco-Durban C, Revilla M, Gonzaloz-Riola J, Rico H (1995) Evaluation of bone density with peripheral quantitative computed tomography in health premenopausal, perimenopausal, and postmenopausal women. Age Ageing 24:447–450

17. Kasamatsu T, Yoshimura N, Morioka S, Sugita K, Hashimoto T (1996) A population survey on bone mineral density in a fishing village in Wakayama prefecture. (Part 1) Distribution of bone mineral density by sex and age based on a representative sample of the community. Nippon Eiseigaku Zasshi 50(6):1084–1092 [Japanese]
18. Hernández-Avila M, Stampfer MJ, Ravnikar VA, Willett WC, Schiff I, Francis M, Longcope C, McKinlay SM, Longcope C (1993) Caffeine and other predictors of bone density among pre- and perimenopausal women. Epidemiology 4(2):128–134
19. Kyriazopoulos P, Trovas G, Charopoulos J, Antonogiannakis E, Galanos A, Lyritis G (2006) Lifestyle factors and forearm bone density in young Greek men. Clin Endocrinol (Oxf) 65(2):234–238
20. Hamdi Kara I, Aydin S, Gemalmaz A, Aktürk Z, Yaman H, Bozdemir N, Kurdak H, Sitmapinar K, Devran Sencar I, Başak O, Akdeniz M, Işildar H, Burgut E, Ozcan S, Akça U, Dağdeviren N, Ungan M (2007) Habitual tea drinking and bone mineral density in postmenopausal Turkish women: investigation of prevalence of postmenopausal osteoporosis in Turkey (IPPOT Study). Int J Vitam Nutr Res 77(6):389–397
21. Shen CL, Wang P, Guerrier J, Yeh JK, Wang JS (2008) Protective effect of green tea polyphenols on bone loss in middle-aged female rats. Osteoporos Int 19(7):979–990
22. Shen CL, Yeh JK, Cao JJ, Tatum OL, Dagda RY, Wang JS (2010) Green tea polyphenols mitigate bone loss of female rats in a chronic inflammation-induced bone loss model. J Nutr Biochem 21(10):968–974
23. Dorchies OM, Wagner S, Vuadens O, Waldhauser K, Buetler TM, Kucera P, Ruegg UT (2006) Green tea extract and its major polyphenol (−)-epigallocatechin gallate improve muscle function in a mouse model for Duchenne muscular dystrophy. Am J Physiol Cell Physiol 290(2):C616–C625
24. Murase T, Haramizu S, Ota N, Hase T (2008) Tea catechin ingestion combined with habitual exercise suppresses the aging-associated decline in physical performance in senescence-accelerated mice. Am J Physiol Regul Integr Comp Physiol 295: R281–R289
25. Woo J, Hong A, Lau E, Lynn H (2007) A randomised controlled trial of Tai Chi and resistance exercise on bone health, muscle strength and balance in community-living elderly people. Age Ageing 36(3):262–268
26. Chen KM, Lin JN, Lin HS, Wu HC, Chen WT, Li CH, Kai Lo S (2008) The effects of a Simplified Tai-Chi Exercise Program (STEP) on the physical health of older adults living in long-term care facilities: a single group design with multiple time points. Int J Nurs Stud 45(4):501–507
27. Shen CL, Williams JS, Chyu MC, Paige RL, Stephens AL, Chauncey KB, Prabhu FR, Ferris LT, Yeh JK (2007) Comparison of the effects of Tai Chi and resistance training on bone metabolism in the elderly: a feasibility study. Am J Chin Med 35(3):369–381
28. Wolfson L, Whipple R, Derby C, Judge J, King M, Amerman P, Schmidt J, Smyers D (1996) Balance and strength training in older adults: intervention gains and Tai Chi maintenance. J Am Geriatr Soc 44(5):498–506
29. Chan K, Qin L, Lau M, Woo J, Au S, Choy W, Lee K, Lee S (2004) A randomized, prospective study of the effects of Tai Chi Chun exercise on bone mineral density in postmenopausal women. Arch Phys Med Rehabil 85(5):717–722
30. Qin L, Au S, Choy W, Leung P, Neff M, Lee K, Lau M, Woo J, Chan K (2002) Regular Tai Chi Chuan exercise may retard bone loss in postmenopausal women: a case–control study. Arch Phys Med Rehabil 83(10):1355–1359
31. Qin L, Choy W, Leung K, Leung PC, Au S, Hung W, Dambacher M, Chan K (2005) Beneficial effects of regular Tai Chi exercise on musculoskeletal system. J Bone Miner Metab 23(2):186–193
32. Kim CJ, Oh KW, Rhee EJ, Kim KH, Jo SK, Jung CH, Won JC, Park CY, Lee WY, Park SW, Kim SW (2009) Relationship between body composition and bone mineral density (BMD) in perimenopausal Korean women. Clin Endocrinol (Oxf) 71(1):18–26
33. Iki M, Kajita E, Dohi Y, Nishino H, Kusaka Y, Tsuchida C, Yamamoto K, Ishii Y (1996) Age, menopause, bone turnover markers and lumbar bone loss in healthy Japanese women. Maturitas 25(1):59–67
34. Sherk VD, Karabulut M, Bemben MG, Bemben DA (2009) Age comparisons of bone density and geometry in men. J Musculoskelet Neuronal Interact 9(4):256–262
35. Marin RV, Pedrosa MA, Moreira-Pfrimer LD, Matsudo SM, Lazaretti-Castro M (2010) Association between lean mass and handgrip strength with bone mineral density in physically active postmenopausal women. J Clin Densitom 13(1):96–101
36. Genaro PS, Pereira GA, Pinheiro MM, Szejnfeld VL, Martini LA (2010) Influence of body composition on bone mass in postmenopausal osteoporotic women. Arch Gerontol Geriatr 51(3):295–298
37. Buetler TM, Renard M, Offord EA, Schneider H, Ruegg UT (2002) Green tea extract decreases muscle necrosis in mdx mice and protects against reactive oxygen species. Am J Clin Nutr 75 (4):749–753
38. Dorchies OM, Wagner S, Buetler TM, Ruegg UT (2009) Protection of dystrophic muscle cells with polyphenols from green tea correlates with improved glutathione balance and increased expression of 67LR, a receptor for (−)-epigallocatechin gallate. Biofactors 35(3):279–294
39. Evans NP, Call JA, Bassaganya-Riera J, Robertson JL, Grange RW (2010) Green tea extract decreases muscle pathology and NF-kappaB immunostaining in regenerating muscle fibers of mdx mice. Clin Nutr 29(3):391–398
40. Call JA, Voelker KA, Wolff AV, McMillan RP, Evans NP, Hulver MW, Talmadge RJ, Grange RW (2008) Endurance capacity in maturing mdx mice is markedly enhanced by combined voluntary wheel running and green tea extract. J Appl Physiol 105(3):923–932
41. Murase T, Haramizu S, Shimotoyodome A, Nagasawa A, Tokimitsu I (2005) Green tea extract improves endurance capacity and increases muscle lipid oxidation in mice. Am J Physiol Regul Inter Comp Physiol 288:R708–R715
42. Xu DQ, Li JX, Hong Y (2006) Effects of long term Tai Chi practice and jogging exercise on muscle strength and endurance in older people. Br J Sports Med 40(1):50–54, discussion 50–54
43. Xu DQ, Li JX, Hong Y (2005) Effect of regular Tai Chi and jogging exercise on neuromuscular reaction in older people. Age Ageing 34(5):439–444
44. Halleen JM, Alatalo SL, Suominen H, Cheng S, Janckila AJ, Väänänen HK (2000) Tartrate-resistant acid phosphatase 5b: a novel serum marker of bone resorption. J Bone Miner Res 15 (7):1337–1345
45. Shen CL, Chyu MC, Yeh JK, Felton CK, Xu KT, Pence BC, Wang JS (2009) Green tea polyphenols and Tai Chi for bone health: designing a placebo-controlled randomized trial. BMC Musculoskelet Disord 10:110
46. Liang SY, Wu WC (1996) Tai Chi Chuan: 24 and 48 postures with martial applications. YMAA Publication Center, Roslindale
47. Godin G, Shephard RJ (1997) Godin leisure time exercise questionnaire. Med Sci Sports Exerc 29:36–38
48. Yesavage JA, Brink TL, Rose TL, Lum O, Huang V, Adey M, Leirer VO (1982) Development and validation of a geriatric depression screening scale: a preliminary report. J Psychiatr Res 17:37–49
49. Folstein MF, Folstein SE, McHugh PR (1975) Mini-mental state. J Psychiatr Res 12(3):189–198
50. Ayotte NW, Stetts DM, Keenan G, Greenway EH (2007) Electromyographical analysis of selected lower extremity muscles during

5 unilateral weight-bearing exercises. J Orthop Sports Phys Ther 37(2):48–55
51. Ekstrom RA, Donatelli RA, Carp KC (2007) Electromygraphic analysis of core trunk, hip, and thigh muscle during 9 rehabilitation exercises. J Orthop Sports Phys Ther 37(12):754–762
52. Ballard JE, McFarland C, Wallace LS, Holiday DB, Roberson G (2004) The effect of 15 weeks of exercise on balance, leg strength, and reduction in falls in 40 women aged 65 to 89 years. J Am Med Womens Assoc 59(4):255–261
53. Vali B, Rao LG, El-Sohemy A (2007) Epigallocatechin-3-gallate increases the formation of mineralized bone nodules by human osteoblast-like cells. J Nutr Biochem 18(5):341–347
54. Chen CH, Ho ML, Chang JK, Hung SH, Wang GJ (2005) Green tea catechin enhances osteogenesis in a bone marrow mesenchymal stem cell line. Osteoporos Int 16(12):2039–2045
55. Tokuda H, Takai S, Matsushima-Nishiwaki R, Akamatsu S, Hanai Y, Hosoi T, Harada A, Ohta T, Kozawa O (2007) (−)-Epigallocatechin gallate enhances prostaglandin F2alpha-induced VEGF synthesis via upregulating SAPK/JNK activation in osteoblasts. J Cell Biochem 100(5):1146–1153
56. Tokuda H, Takai S, Hanai Y, Matsushima-Nishiwaki R, Hosoi T, Harada A, Ohta T, Kozawa O (2007) (−)-Epigallocatechin gallate suppresses endothelin-1-induced interleukin-6 synthesis in osteoblasts: inhibition of p44/p42 MAP kinase activation. FEBS Lett 581(7):1311–1316
57. Kirsteins AE, Dietz F, Hwang SM (1992) Evaluating the safety and potential use of a weight-bearing exercise, Tai-Chi Chuan, for rheumatoid arthritis patients. Am J Phys Med Rehabil 70(3):136–141
58. Karabulut M, Bemben DA, Sherk VD, Anderson MA, Abe T, Bemben MG (2011) Effects of high intensity resistance training and low-intensity resistance training with vascular restriction on bone markers in older men. Eur J Appl Physiol [Epub ahead of print]
59. Whipple TJ, Le BH, Demers LM, Chinchilli VM, Petit MA, Sharkey N, Williams NI (2004) Acute effects of moderate intensity resistance exercise on bone cell activity. Int J Sports Med 25(7):496–501
60. Sartorio A, Lafortuna C, Capodaglio P, Vangeli V, Narici MV, Faglia G (2001) Effects of a 16-week progressive high-intensity strength training (HIST) on indexes of bone turnover in men over 65 years: a randomized controlled study. J Endocrinol Invest 24 (11):882–886
61. Zittermann A, Geppert J, Baier S, Zehn N, Gouni-Berthold I, Berthold HK, Reinsberg J, Stehle P (2004) Short-term effects of high soy supplementation on sex hormones, bone markers, and lipid parameters in young female adults. Eur J Nutr 43(2):100–108
62. Viljakainen HT, Väisänen M, Kemi V, Rikkonen T, Kröger H, Laitinen EK, Rita H, Lamberg-Allardt C (2009) Wintertime vitamin D supplementation inhibits seasonal variation of calcitropic hormones and maintains bone turnover in healthy men. J Bone Miner Res 24(2):346–352
63. Miller LJ 3rd, Crowson CS, O'Fallon WM, Wahner HW, Riggis BL (2003) Relative contributions of bone density, bone turnover, and clinical risk factors to long-term fracture prediction. J Bone Miner Res 18(2):312–318
64. Flemiing KH, Heimbach JT (1994) Consumption of calcium in the U.S.: food sources and intake levels. J Nutr 124(8 Suppl):1426S–1430S
65. Fulgoni V 3rd, Nicholls J, Reed A, Buckley R, Kafer K, Huth P, DiRienzo D, Miller GD (2007) Dairy consumption and related nutrient intake in African-American adults and children in the United States: continuing survey of food intakes by individuals 1994–1996, 1998, and the National Health And Nutrition Examination Survey 1999–2000. J Am Diet Assoc 107(2):256–264
66. Kanders B, Dempster DW, Lindsay R (1988) Interaction of calcium nutrition and physical activity on bone mass in young women. J Bone Miner Res 3(2):145–149
67. Kemmler W, Lauber D, Weineck J, Hensen J, Kalender W, Engelke K (2004) Benefits of 2 years of intense exercise on bone density, physical fitness, and blood lipids in early postmenopausal osteopenic women: results of the Erlangen Fitness Osteoporosis Prevention Study (EFOPS). Arch Intern Med 164(10):1084–1091
68. Jilka RL, O'Brien CA, Bartell SM, Weinstein RS, Manolagas SC (2010) Continuous elevation of PTH increases the number of osteoblasts via both osteoclast-dependent and -independent mechanisms. J Bone Miner Res 25(11):2427–2437
69. Dempster DW, Cosman F, Parisien M, Shen V, Lindsay R (1993) Anabolic actions of parathyroid hormone on bone. Endocr Rev 14:690–709
70. Canalis E, Hock JM, Raisz LG (1994) Anabolic and catabolic effects of parathyroid hormone on bone and interactions with growth factors. In: Bilezikian JP, Marcus R, Levine MA (eds) The parathyroids: basic and clinical concepts. Raven, New York, pp 65–82
71. Thorsen K, Kristoffersson A, Hultdin J, Lorentzon R (1997) Effect of moderate endurance exercise on calcium, parathyroid hormone, and markers of bone metabolism in young women. Calcif Tissue Int 60:16–20
72. Brahm H, Piehl-Aulin K, Saltin B, Ljunghall S (1997) Net fluxes over working thigh of hormones, growth factors and biomarkers of bone metabolism during short lasting dynamic exercise. Calcif Tissue Int 60(2):175–180
73. Maltais ML, Desroches J, Dionne IJ (2009) Changes in muscle mass and strength after menopause. J Musculoskelet Neuronal Interact 9(4):186–197
74. Wu G, Liu W, Hitt J, Millon D (2004) Spatial, temporal and muscle action patterns of Tai Chi gait. J Electromyogr Kinesiol 14 (3):343–354
75. Wu G (2008) Muscle action pattern and knee extensor strength of older Tai Chi exercisers. Med Sport Sci 52:30–39
76. Tseng SC, Liu W, Finley M, McQuade K (2007) Muscle activation profiles about the knee during Tai-Chi stepping movement compared to the normal gait step. J Electromyogr Kinesiol 17 (3):372–380
